# Civilian public sector employment as a long-run outcome of military conscription

**DOI:** 10.1073/pnas.1908983116

**Published:** 2019-10-08

**Authors:** Tim Johnson, Dalton Conley

**Affiliations:** ^a^Atkinson Graduate School of Management, Willamette University, Salem, OR 97301;; ^b^Center for Governance and Public Policy Research, Willamette University, Salem, OR 97301;; ^c^Department of Sociology, Princeton University, Princeton, NJ 08544;; ^d^National Bureau of Economic Research, Cambridge, MA 02138

**Keywords:** employment, labor market, public service, state development, military veterans

## Abstract

Since at least T. H. Marshall, scholars have recognized military service as a form of sacrifice that warrants compensation from the state. War-widow pensions, expansion of the franchise, and subsidized higher education are all examples of rights and benefits “bestowed” in return for wartime mobilization. Similarly, in the United States, governments have hired veterans preferentially for civilian public jobs as recompense for active military service. Although oft overlooked, those policies seem influential: the percentage of job holders identifying as veterans in the civilian US executive branch exceeds the proportion in the wider population by several multiples. This century-old pattern suggests another way that wartime mobilization has influenced the state. Yet, efforts to understand it have struggled to rule out the possibility that those who serve in the armed forces are predisposed to work for the state in both military and civilian capacities. Here, we rule out this possibility by examining whether birthdates randomly called for induction in the Vietnam-Era Selective Service Lotteries (VSSL) appear disproportionately in the population of nonsensitive personnel records of the civilian US executive branch. We find that birthdates called for induction appear with unusually high frequency among employees who were draft eligible and at risk for induction but not among other employees. This finding suggests a treatment effect from military service, thus dovetailing with the hypothesis that wartime mobilization has substantially and continually influenced who works in the contemporary administrative state.

## Significance

Wartime mobilization shapes state development, since veterans also display high rates of civilian public employment. Such a pattern could result from a treatment effect of military service—likely resulting from government programs that institute veterans hiring preferences. Alternatively, veterans may be temperamentally predisposed to prefer public employment. We rule out this latter self-selection possibility by examining whether birthdates randomly called for induction in the Vietnam-Era Selective Service Lotteries appear disproportionately in the population of nonsensitive personnel records of the civilian US executive branch. We find that birthdates called for induction appear with disproportionately high frequency among draft-eligible employees at risk of induction. Net of selection, military service affects entry into public sector employment, and thus, wartime mobilization continues to influence who works in the administrative state.

Research argues that early states emerged from efforts to muster resources for war (1), and the modern welfare state shares a similar origin. At least since T. H. Marshall (2) identified wartime sacrifice as a basis for making claims on the state, scholars have documented how states have extended rights, privileges, and subsidies both to encourage wartime sacrifice (3, 4) and to compensate for it (5). Expansion of the franchise (4), commitments to procedural fairness (3), pensions for war widows (5), and subsidized higher education (6), to name a few examples, all reflect state efforts to elicit or reward sacrifice in times of war. Together, these mechanisms of compensation formed the modern welfare state, and their amplification in recent decades for military personnel and their families has transformed the armed forces into an enterprise with both martial and welfare purposes (7).

Yet, while these policies had a foundational role in the formation of the contemporary welfare state, an often-ignored form of compensation for military sacrifice may continue to shape the contemporary state. For over a century, the US Federal Government and various state governments have hired military veterans preferentially for public jobs (8, 9). These policies echo similar practices in early states (10–12), and research claims that these policies explain why veterans staff over 25% of US executive branch positions, despite constituting less than 10% of the US population (8, 13). Such figures suggest a marked, continual influence of wartime compensation on the state: compensation for military participation may open a path to civilian public service that veterans are particularly inclined to traverse. Indeed, evidence indicates that federal government jobs offer a wage premium (14); thus, with preferential hiring in place, military service amounts to the assignment of a voucher giving greater access to a desirable asset.

The overrepresentation of military veterans among civilian employees of the government also may stem from the bundle of treatment effects that military service potentially assigns to job seekers. For instance, veterans might acclimate to the organizational structure of the military, which shares features with the civilian public bureaucracy, thus making them even more inclined to take advantage of the opportunities made available via preferential hiring (9). Perhaps less optimistically, veterans might lack experience in the civilian labor market, thus impeding their efforts to find private sector work and causing them to gravitate toward public jobs in which preferential hiring compensates for their lack of civilian labor market experience. Military service might increase patriotism, thus inspiring civilian public service after discharge. Any one of these mechanisms might underlie the effect of military service—which is indelibly linked with the receipt of federal preferential hiring benefits—on entry into public employment.

Estimating such treatment effects of military service on choice of employment sector is complicated by selection dynamics. Namely, those who serve in the armed forces might be predisposed to enter both military and civilian public service. For instance, they might prefer the comparative stability of public employment, exhibit consistently high levels of patriotism throughout their lives, or possess a taste for working in large enterprises. That is, veterans’ rates of entry into public jobs might merely reflect confounding, not the effect of military service and the eligibility for preferential hiring that is linked with it. Accordingly, to understand how the assignment of military service in the presence of preferential hiring influences entry into the executive branch and thus, to gain insight into the way that wartime mobilization might continue to affect the state, research must eliminate possible confounders and examine how exogenous variation in military service affects the ranks of the civilian public service. If exogenously assigned military service does not covary with entry into public employment, then preservice factors—not military service in the presence of preferential hiring—likely drive veterans toward public jobs.

Existing evidence, indeed, is inconsistent in its assessment of whether exogenous variation in military service affects the likelihood of subsequent public employment. Angrist and Chen (15)—in a study of the late-life, labor market experiences of men eligible for the Vietnam-Era Selective Service Lotteries (VSSL)—discovered that men’s chances of working in the public sector increased with their risk of military induction thanks to having their draft number called. Green et al. (16), however, in a study of whether the VSSL shaped draftees’ political attitudes, found—in a separate sample—that the estimated effect of lottery-induced military service on public sector employment was smaller than its SE—i.e., a “null” effect. Unfortunately, the different methods of each study—an instrumental variable analysis (15) and an intent-to-treat analysis (16)—prevent direct comparison of the studies’ discrepant effect estimates. Furthermore, both studies investigate a sample; thus, their divergent findings could be attributable merely to sampling error: chance sampling variation could result in datasets that, say, over- or underrepresent study participants from geographical areas with more generous (or less generous) state and local preferential hiring policies. Also, by grouping federal and lower-level employment into the same indicator of public employment, these studies mask potentially heterogeneous treatment effects that result from the systematic variation in treatment dosage across governmental jurisdictions.

The study that we report in this paper overcomes these problems by deploying the population of data for one level of government that is subject to a single set of preferential hiring policies. Specifically, the study examines whether birthdates called for induction in the VSSL appear with disproportionately high frequency in the population of US executive branch employees who were draft eligible and therefore at risk for induction. This analysis tests whether the assignment of a higher probability of military induction increases later rates of entry into civilian federal jobs, thereby shedding light on whether wartime mobilization not only played an important role in forming the state, but, also, continuously shaped it by populating its civilian ranks.

## Methods

The VSSL provides an exogenous source of variation in draft-eligible men’s risk of military service (17). We parrot past studies’ description of the VSSL here (ref. 18, footnote 6). In the VSSL, the US Selective Service System printed calendar dates on paper strips, placed the strips in small pods, put these pods in a receptacle, and drew pods one-by-one, assigning numbers sequentially to each month–day combination until all received numbers. These “Random Sequence Numbers” (RSNs) identified when draft-eligible men reported for military induction in the following year. Men with RSN 1 reported first, those with RSN 2 reported second, and this process continued until the RSN reached the year’s “Administrative Processing Number” (APN). The APN was set before induction; it equaled 195 for the 1969 lottery, 125 in the second year, and 95 every year until the final lottery in 1975. After the VSSL’s first 3 years, numbers were assigned but not called.

Knowing these procedures, one of the authors (T.J.) filed a Freedom of Information Act request with the US Office of Personnel Management (OPM) for all birthdates in the Central Personnel Data File (CPDF)—the population of official personnel data for nondefense and nonpostal agencies of the US federal executive branch. These data include all employees, whether they work a full-time or part-time schedule, across all agencies and occupations included in the CPDF. Officials at the OPM supplied a count of the total number of males and females, respectively, holding each birthdate in the monthly archived population of personnel data from June 2011 (the first month in which full birthdates were systematically collected) to March 2016 (the most recent month of data available at the time of the request). Rare birthdates held by less than 5 employees had their counts set to 0 to protect employee privacy, but no such birthdates appeared in the data studied in the analyses that we report below. Indeed, due to the sensitive nature of birthdates, the OPM did not release additional variables with the data that would allow us to discern the agencies or occupations of employees or personal information, such as the employees’ race. However, in *SI Appendix*, we report summary statistics from a publicly available, nonsensitive version of the CPDF that, albeit void of birthdates, includes detailed information about the workplace and personal characteristics of veterans’ preference recipients and nonrecipients in the VSSL birth cohorts. All data and computer code used in these analyses are available via the web addresses provided in the data deposition footnote.

The time period of the data studied here makes them roughly comparable with those of Angrist and Chen (15) and Green et al. (16), which both reported the effect of the VSSL on later-life public employment. Given that this time period extends decades beyond the VSSL, estimates from our research design inevitably reflect patterns of retirement and mortality caused by draft-eligible men’s lottery numbers. However, due to our study’s aim of examining the lasting effect of military service on the administrative state, such effects should be a part of our estimation: if low lottery numbers drove individuals to work in the federal government later into their lives or to retire earlier than their colleagues with high lottery numbers, then those phenomena ultimately shaped how military service affected employment in the federal government over the long term, which is the phenomenon that we seek to understand.

We subset the data to include birthdates from 1950 to 1956, thereby focusing exclusively on birth cohorts turning 19 y old during the VSSL. Consistent with the past literature (19), this decision enhances the precision and interpretation of effect estimates by focusing the investigation on a homogenous population. Men born as early as 1944 remained eligible for the first lottery draw if they had avoided conscription previously, yet the fact that they had done so increased the chance that they could avoid conscription subsequently (e.g., due to medical reasons). Thus, their inclusion would add noise to our effect estimates via treatment effect heterogeneity. Furthermore, older birth cohorts lack a clear counterfactual: the attributes that made them eligible into their early 20s would need to be reflected in observations with which they are compared, yet we lack data about these diverse, unobservable attributes. As a result, estimates drawn from older birth cohorts would resist interpretation. For these reasons, we follow the past literature (18, 19) and solely study the 1950 to 1956 birth cohorts.

To study these data, we used the research design of Conley and Heerwig (20). We calculated $P_{S,t}$—that is, the proportion of employees, *P*, identifying with sex, *S*, born in year, *t*, whose birthdates were assigned an RSN ≤ APN. If the VSSL influenced entry into civilian executive branch employment, one would expect $P_{M,1950}>P_{F,1950},P_{M,1951}>P_{F,1951}$, and $P_{M,1952}>P_{F,1952}$. Moreover, given that no numbers were called after 1952, one would also expect $P_{M,1953}≅P_{F,1953},P_{M,1954}≅P_{F,1954},P_{M,1955}≅P_{F,1955}$, and $P_{M,1956}≅P_{F,1956}$. As an additional counterfactual, we calculated $P_{E,t}$—that is, the theoretically expected proportion of employees holding birthdates assigned an RSN ≤ APN for either sex, *E*, in year, *t*, if birthdates are uniformly distributed across the year (20). For instance, $P_{E,1950}$ = 195/365 = 0.5328, because 1950 had 365 possible birthdates (it was not a leap year), and the APN equaled 195 for the 1950 birth cohort. Accordingly, we expect $P_{M,1950}>P_{E,1950}$, $P_{M,1951}>P_{E,1951}$, $P_{M,1952}>P_{E,1952}$, $P_{M,1953}≅P_{E,1953}$, $P_{M,1954}≅P_{E,1954}$, $P_{M,1955}≅P_{E,1955}$, and $P_{M,1956}≅P_{E,1956}$.

## Results

Results dovetail with these expectations (Table 1). In the 1950 to 1952 birth cohorts, the proportion of male birthdates assigned an RSN ≤ APN is markedly higher than the corresponding proportions for birthdates held by females. Furthermore, in those birth cohorts, the female proportions more closely approximate the theoretically expected proportion of birthdates assigned an RSN ≤ APN than do the corresponding male proportions. In the 1953 to 1956 birth cohorts, the proportions of male birthdates assigned an RSN ≤ APN differ less noticeably from the corresponding proportions for birthdates held by females. Also, the male proportions for the 1953 to 1956 birth cohorts approximate their corresponding, theoretically expected proportion of birthdates more closely than do the male proportions for the 1950 to 1952 cohorts.

**Table 1.** Proportion of employee birthdates assigned lottery numbers called for induction

Birth year (*t*)	I. Proportion males (*M*): $P_{M,t}$	II. Proportion females (*F*): $P_{F,t}$	III. Expected proportion (*E*): $P_{E,t}$	IV. Difference I – II: $D_{MF,t}=$ $P_{M,t}-P_{F,t}$	V. Difference I – III: $D_{ME,t}=$ $P_{M,t}-P_{E,t}$	VI. Difference II – III: $D_{FE,t}=$ $P_{F,t}-P_{E,t}$
1950	0.5801	0.5449	0.5328	0.0352	0.0473	0.0121
1951	0.3765	0.3422	0.3425	0.0343	0.0340	−0.0003
1952	0.2987	0.2570	0.2596	0.0417	0.0391	−0.0026
1953	0.2684	0.2559	0.2603	0.0125	0.0081	−0.0044
1954	0.2609	0.2669	0.2603	−0.0060	0.0006	0.0066
1955	0.2589	0.2595	0.2603	−0.0006	−0.0013	−0.0008
1956	0.2593	0.2592	0.2596	0.0001	−0.0003	−0.0004

Values are rounded.

**Fig. 1.**
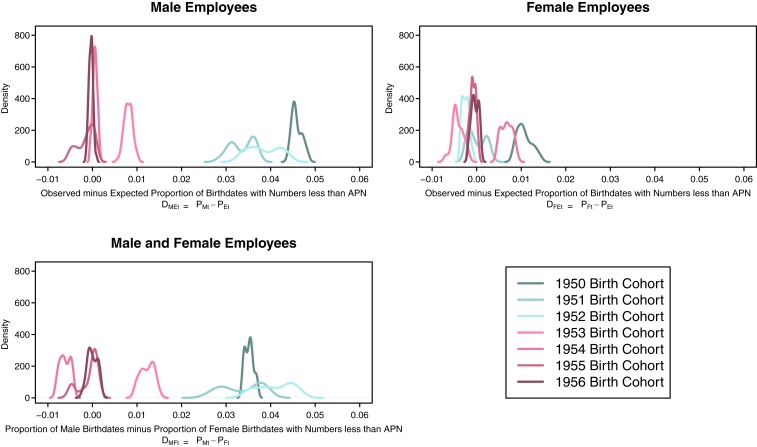
Distribution of calculated effects across all months of the CPDF, June 2011 to March 2016. This figure presents kernel density plots showing the distribution of $D_{ME,t}=P_{M,t}-P_{E,t}$ (*Upper Left*), $D_{FE,t}=P_{F,t}-P_{E,t}$ (*Upper Right*), and $D_{MF,t}=P_{M,t}-P_{F,t}$ (*Lower Left*), by annual birth cohort, calculated using each monthly archived version of the CPDF. In *Upper Left*, distributions of $D_{ME,t}$ rest closer to 0 for birth cohorts with numbers that were not called for induction (1953 to 1956) than for male birth cohorts that experienced the call of lottery numbers (1950 to 1952). Distributions of $D_{FE,t}$ congregate near 0 for all birth cohorts (*Upper Right*), which is consistent with the fact that females were not eligible for the draft lotteries. The distributions of $D_{MF,t}$ diverge substantially from 0 for the 1950 to 1952 birth cohorts but not for the 1953 to 1956 birth cohorts (*Lower Left*).

These findings arise from an analysis that pools monthly archived sets of the CPDF. Repeating the analysis on each monthly archived CPDF does not lead to different conclusions. Fig. 1 presents kernel density plots showing the distribution of $D_{ME,t}=P_{M,t}-P_{E,t}$ (Fig. 1, *Upper Left*), $D_{FE,t}=P_{F,t}-P_{E,t}$ (Fig. 1, *Upper Right*), and $D_{MF,t}=P_{M,t}-P_{F,t}$ (Fig. 1, *Lower Left*) by annual birth cohort when repeating the analysis on each monthly archived version of the CPDF. Across panels, the distribution of these differences yields substantively comparable findings to the pooled results: birthdates assigned an RSN ≤ APN appear with disproportionately high frequency solely among draft-eligible male employees at risk for induction.

These results indicate a link between lottery numbers and civilian public employment. However, they rely heavily on the assumption that the APN cutoff value was administered accurately and uniformly. That said, errors in the administration of the induction call may have meant that adherence to the APN cutoff was more variable in practice than was stated in policy. Accordingly, we examined whether the substantive interpretation of the findings—namely, that the VSSL influenced the ranks of executive branch employment—remained valid even when the APN cutoff did not figure prominently in the study design. To do so, we examined the relationship between the numerical value of RSNs assigned to federal employees and the number of employees holding the birthdates associated with those RSNs.

Given that low RSNs were called for induction first and given that the military’s need for personnel diminished as the induction call proceeded, one would expect—if the VSSL influenced rates of federal employment—a decreasing relationship between the value of RSNs and the number of employees holding birthdates assigned those RSNs solely in data concerning men born from 1950 to 1952. However, among females as well as males in the placebo cohorts, one would expect to find a null relationship between the value of employees’ lottery numbers and the number of employees holding the birthdates to which those RSNs were assigned.

**Fig. 2.**
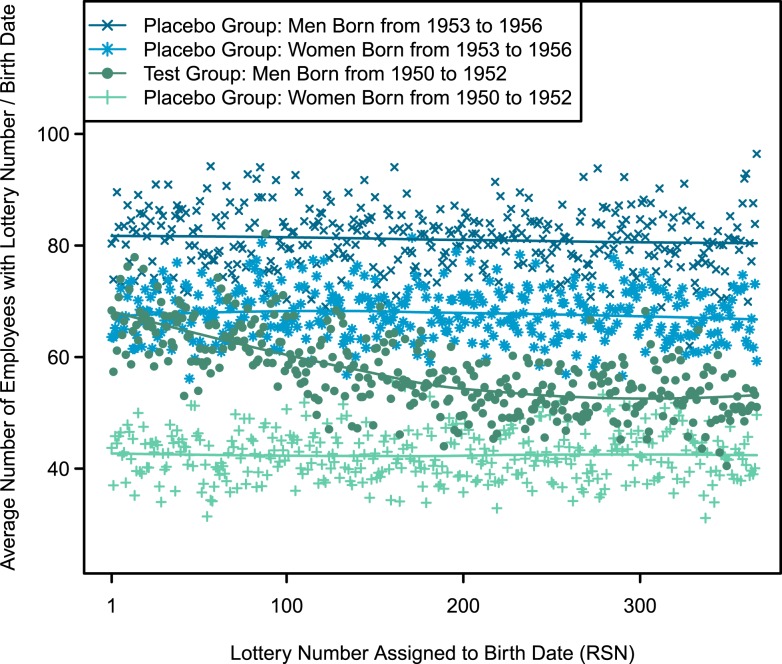
Numerical value of lottery numbers and the average number of employees holding them. This figure plots the average number of employees holding a given lottery number by the numerical value of that lottery number (RSN). Among female employees in the studied birth cohorts or male employees born from 1953 to 1956, the average number of employees holding a given birthdate does not appear to vary systematically with the birthdate’s assigned lottery number. However, among male employees born from 1950 to 1952, a negative relationship appears between lottery numbers and the mean number of employees holding them. This negative relationship declines steeply over the first 200 lottery numbers and then, begins to flatten.

As a first step in testing those expectations, Fig. 2 shows a plot of the average number of employees holding a given lottery number and the value of that lottery number. Only among male employees born from 1950 to 1952 does a negative relationship appear between lottery numbers and the mean number of employees holding those RSNs. Moreover, among male employees born from 1950 to 1952, this negative relationship appears strong over the first 200 lottery numbers; then, it flattens out thereafter. This trend reflects the fact that the highest APN capped the call for induction at 195: if exposure to military service influences rates of entering the federal civilian bureaucracy, one would not expect the value of lottery numbers beyond this highest APN to influence the number of federal employees holding birthdates associated with those RSNs.

**Fig. 3.**
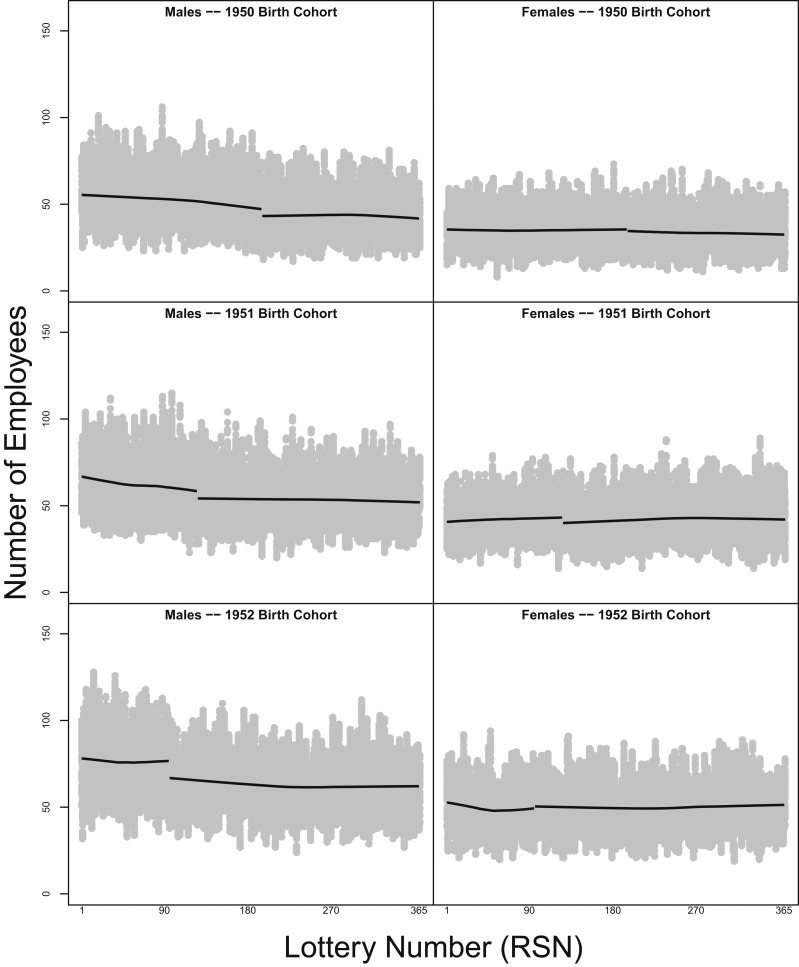
Value of lottery numbers and the raw number of employees holding them: 1950 to 1952 birth cohorts. Panels present raw data—from monthly archived versions of the CPDF—concerning federal employees distinguished by their sex and annual birth cohort. The numerical value of lottery numbers appears on the horizontal axis, and the raw number of employees holding those numbers appears on the vertical axis. Two smoothers depict estimates of employees with lottery numbers at or below the APN and employees with lottery numbers above the APN. *Left* shows that the number of male employees declines as the values of their lottery numbers approach the APN. No such pronounced, decreasing trend appears in *Right*, which consists of data concerning female employees from the 1950 to 1952 birth cohorts.

**Fig. 4.**
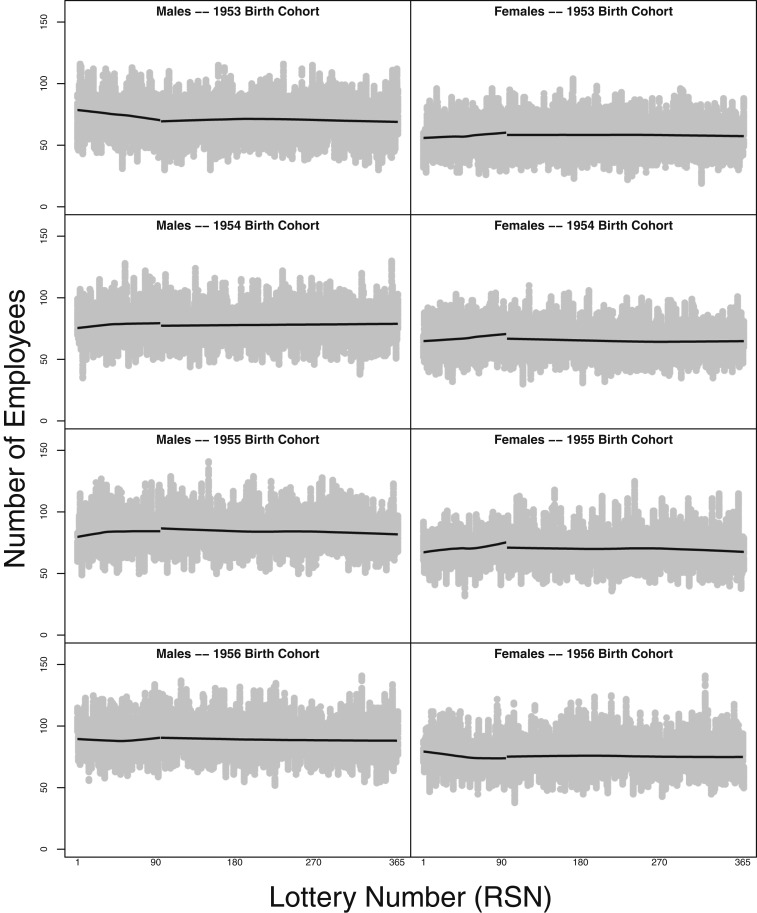
Value of lottery numbers and the raw number of employees holding them: 1953 to 1956 birth cohorts. Panels present raw data—from monthly archived versions of the CPDF—concerning federal employees distinguished by their sex and annual birth cohort as in Fig. 3. Two smoothers portray estimates of employees with lottery numbers at or below the APN and employees with lottery numbers above the APN.

Fig. 3 shows that a similar trend appears in annual birth cohorts, and it displays the discontinuity that appears at the APN cutoff in each year. That is, in each panel in Figs. 3 and 4, we present raw data—from monthly archived versions of the CPDF—concerning federal employees distinguished by their sex and annual birth cohort. The value of lottery numbers appears on the horizontal axis, and the raw number of employees holding those values appears on the vertical axis. In each panel, we display 2 smoothers estimated on employees with lottery numbers at or below the APN and employees with lottery numbers above the APN, respectively. Fig. 3, *Left* displays data for male employees born from 1950 to 1952; in each panel, the number of male employees declines as the values of their lottery numbers approach the APN. No such pronounced, decreasing trend appears in Fig. 3, *Right*, which consists of data concerning female employees from the 1950 to 1952 birth cohorts. Furthermore, across Fig. 3, *Right*, a noticeable discontinuity appears at the APN, thus indicating a drop in the number of employees holding lottery numbers greater than the APN. No other panels show this drop at the APN cutoff as vividly. Although male employees born in 1953 (Fig. 4) exhibit a decreasing relationship between lottery numbers and the number of employees to the left of the APN (note that APN = 95 in 1953),* no noteworthy discontinuity appears at the APN’s value. However, data concerning female employees born in 1951, 1954, and 1955 exhibit a pronounced discontinuity at the APN, but the smoother slightly increases as it approaches the APN value from the left, indicating that higher lottery numbers appeared more frequently in the data. Overall, in these panels concerning placebo cohorts, the data appear to exhibit a null relationship, suggesting that birthdates associated with lottery numbers at or below the APN do not appear with disproportionate frequency in the placebo cohorts. The findings indicate that a study design that puts less emphasis on the APN cutoff still provides evidence suggesting that the VSSL influenced the ranks of the civilian executive branch.

## Conclusion

Efforts to mobilize martial resources sparked the development of early states (1), and methods of repaying and inspiring wartime sacrifice resulted in the modern welfare state (5). Although these germinal effects of war on the state have received considerable scrutiny, the continual effect of wartime mobilization on who works in the contemporary state has received less attention, although long-standing evidence indicates unusually high rates of public sector employment among veterans—especially in the US executive branch (8, 12). This pattern, however, could result from a predisposition that drives individuals to work for the state in both military and civilian capacities, thus calling into question the notion that wartime compensation continually affects the state by influencing who works for it. To rule out that possibility, we examined whether birthdates randomly called for induction in the VSSL appeared excessively in the population of nonsensitive personnel records of the civilian, nondefense, and nonpostal US executive branch. We found that draft-eligible birthdates did indeed appear with unusually high frequency among male employees but not among female employees. Our findings dovetail with the notion that exogenously imposed military service increases rates of entering civilian US executive branch employment, thus supporting the hypothesis that wartime mobilization has influenced the contemporary administrative state continually and, for the federal government, reconciling discrepant results in the past literature (15, 16).

These results raise the possibility that large-scale military mustering in times of war or the maintenance of large peacetime armies may have important long-term consequences on the composition of public and private sector labor markets. Research indicates that veterans’ high level of entering public employment alters the demographic representation of government workforces (8). In the US federal executive branch workforce, veterans’ preference appears to have increased the share of jobs staffed by individuals identifying as white males and to a lesser extent, individuals identifying as black males. In *SI Appendix*, we offer evidence suggesting that these same trends occurred within the draft-eligible cohorts studied herein. Specifically, in *SI Appendix*, we show that the average number of white males across the 1950 to 1952 birth cohorts substantially exceeded the mean number of white males across the 1953 to 1956 birth cohorts, and this trend appears more pronounced when comparing the average number of white males benefitting from veterans’ preference across the 2 sets of birth cohorts.

If preferential hiring serves as the mechanism attracting veterans to government employment, then our findings also raise policy questions about the performance of the US Federal Government workforce. Preferential hiring putatively weakens the inspection that veterans experience during the hiring process, thus potentially letting lower-performing workers into the federal workforce (8, 9). Military service, however, might prepare individuals for roles in the civilian public sector, thus making preferential hiring benefits a valid, albeit unintentional, cue of a job candidate’s prospective performance (9). Empirical studies have tested these possibilities by examining whether military veterans climb to higher levels of responsibility or pay—surrogates for performance—at the same rate as nonveterans and have found mixed results (8, 9, 21). *SI Appendix* reports analyses in which we replicate the methods of these studies on the VSSL birth cohorts and find little evidence of the pay differences that one would expect if the 2 groups of employees exhibited markedly different performance. Those findings, however, are preliminary, and further attention ought to be paid to this important policy implication.

A final implication relates to whether veterans in public employment staff positions that shape policy and whether veterans might be inclined to drive policy in particular ideological directions. As reported in *SI Appendix*, between 8 and 13% of veterans’ preference recipients in the VSSL birth cohorts staffed supervisory positions in which they led or directed the work of other federal employees—values that do not differ considerably from those of nonrecipients of preference. Such authority raises the possibility of policy influence, yet research does not currently offer detailed information about the ideological leanings—if any—of veterans in the public sector. Investigating those political preferences and the policy authority of veterans in the US Federal Government promises to shed light on a salient potential policy implication of our study.

With those implications recognized, some limitations of this study should be noted. Here, we focused on only a single slice of the public sector—the US federal executive branch. With roughly 2.06 million employees, the nondefense and nonpostal executive branch of the US Federal Government is the largest single civilian, public sector employer in the United States (22). In comparison, the US federal judicial branch contains only 32,711 employees, and the legislative branch employs 30,103 workers (22). The nonpostal and nondefense executive branch also employs about half a million more workers than the total number of state and local employees in California, which ranks as the state with the most such workers, but that portion of the federal executive branch would still amount to only 14% of the 14,617,399 individuals working in state and local governments across the United States (23). Thus, readers should recognize that the results reported here, although comprehensive for a given segment of the public sector, do not tell the full story of how the VSSL affected public sector employment across the United States.

In fact, if we assume that the treatment effect of Vietnam Era military service compelled veterans to seek other forms of public service (such as at the state or local level), then the overall impact on labor markets is proportionally greater than what we have shown here. However, given that the draft lottery induced military service into the US Army and not, say, state National Guard units, its treatment may have induced veterans to seek federal employment to a greater extent than local- or state-level public sector service. We think future research using the draft lottery is warranted to investigate the treatment effect of military induction on all levels of public sector employment as well as that in the private sector (particular occupations, job categories, or agencies may disproportionately drive the effects that we observe). Likewise, beyond studies of earnings, the impact of draft status on private sector job type is also worthy of study.

A second limitation of this study is our inability, with these data, to distinguish the mechanism for the treatment effect that we observe. While our study design effectively rules out simultaneous selection into the military and civilian public sectors, we cannot discern the nature of the effect. Such overrepresentation in the public sector after military service may stem from the preferential hiring of veterans, increased feelings of patriotism, a loss of competitiveness in the private sector due to missing private labor market experience, a sense of entitlement to public sector “spoils” after serving in the military, socialization to organizational structures common across the military and civilian public bureaucracies, or any number of other dynamics. Adjudicating between these mechanisms will further illuminate the findings of this study, which show a prominent way in which mobilization for war continues to affect the state.

## Supplementary Material

Supplementary File

## Footnotes

*In both this analysis and in the findings reported earlier in the text, numbers in the VSSL seem to have had a slight effect on the executive branch employment of men from the 1953 birth cohort. Given that 1953 was the first year in which VSSL numbers were not called for induction, it could be that men with low numbers volunteered for forms of military service that they perceived as less risky in anticipation of their numbers being called. This phenomenon would have resulted in exposure to military service and the acquisition of veteran status, thus remaining consistent with the possibility that personnel policies incentivizing veterans to join the executive branch are responsible for veterans’ overrepresentation in public jobs. In any event, the modest effects for that cohort—compared with the comparable findings for the 1950 to 1952 male cohorts—suggest that, even if such a phenomenon took place, its substantive import is relatively limited.
