# Supplementary material for: Civilian public sector employment as a long-run outcome of military conscription
Source: Proc Natl Acad Sci U S A. 2019 Oct 8;116(43):21456–62. doi: 10.1073/pnas.1908983116 (PMC6815180; doi:10.1073/pnas.1908983116)
Supplement: Supplementary File [file pnas.1908983116.sapp.pdf]

PNAS
www.pnas.org

Supplementary Information for:

Civilian public sector employment as a long-run outcome of military conscription

Tim Johnson, Willamette University
Dalton Conley, Princeton University

Correspondence to TJ (tjohnson@willamette.edu) or DC (dconley@princeton.edu)

Data and computer code used in the analyses reported in this investigation are available at the following links:
www.willamette.edu/~tjohnson/johnson_conley_replication_materials_PNAS_main_text.zip
www.willamette.edu/~tjohnson/johnson_conley_replication_materials_PNAS_supp_info.zip

This PDF file includes:

Contents	Page
1. Overview of the supplementary materials	S1
2. Racial identities of employees receiving veterans' preference	S2
3. Attributes of jobs staffed by veterans' preference recipients upon entry	S7
4. Effect on the productivity or performance of the civilian federal government	S8
5. Position attributes and productivity by veterans' preference status and race	S9
6. Replication of sections (1) – (4) excluding employees of the Dept. of Defense	S9
7. Figure S1	S11
8. Figure S2	S12
9. Figure S3	S13
10. Figure S4	S14
11. Figure S5	S15
12. Figure S6	S16
13. Figure S7	S17
14. Figure S8	S18
15. Figure S9	S19
16. Figure S10	S20
17. Table S1	S21
18. Table S2	S22
19. Table S3	S23
20. Table S4	S24
21. Table S5	S25
22. Table S6	S26
23. Table S7	S27
24. Table S8	S28
25. Table S9	S29
26. Table S10	S30
27. Table S11	S31
28. Table S12	S32
29. Table S13	S33
30. Table S14	S34
31. Table S15	S35
32. Table S16	S36
33. Table S17	S37
34. Table S18	S38
35. Table S19	S39
36. Table S20	S40
37. References cited in the supplementary materials	S41

1. Overview of the supplementary materials

In the main text, we report results indicating that birthdates called in the U.S. Vietnam Selective Service Lotteries (VSSL) appear disproportionately in the personnel records of the civilian U.S. federal executive branch. These results suggest that military service influences individuals' likelihood of entering federal employment—a result consistent with the assumptions of past research and correlational analyses (*1*). Our results do not speak to the mechanisms driving the phenomenon or the policy implications of it; a detailed analysis of those mechanisms and implications rests beyond the scope of our investigation. However, simple analyses can lay the foundation for such an inquiry, thus we perform those analyses and report their results in these supplementary materials.

The evidence we report herein derives from the analysis of data different from those reported in the main text. The data reported in the main text were aggregate counts of the number of male and female U.S. federal employees holding certain birthdates. Due to the sensitive nature of U.S. federal employee records containing birthdates, those data cannot be merged with other variables and we do not possess individual-level records containing birthdates that would allow us to study, say, the racial identities of employees by birthdate or the distribution of birthdates across agencies. Accordingly, the data used in these supplementary materials lack full birthdate information, but they provide the complete population of individual-level data—sans sensitive variables—from the U.S. Central Personnel Data File (CPDF), maintained by the Office of Personnel Management (OPM), from 1973 to 1997. These data include detailed information about employees' racial identities, occupations, supervisory duties, veterans' preference status, and agency of employment—not to mention dozens of other variables.

One of the authors (TJ) obtained these data from the National Archives and Records Administration (NARA). Comparable data sets obtained via the Freedom of Information Act with more-recent years of data (see *2*) lack some of the variables we study here (rules for the release of information from the CPDF differ apparently between OPM and NARA). Accordingly, we consciously trade recency, which our data lack, for detail, which our data possess. This trade is beneficial because of our interest in cohorts of employees eligible for the VSSL. The early years of those employees' careers took place during the years of data we study in these supplementary materials. Thus, the fact that the data are dated make them, in fact, more appropriate for our investigation.

Indeed, the first mechanism that we seek to study in these supplementary materials relates to the possibility that veterans' preference provides an avenue of entry into the civilian federal workforce for individuals who face discrimination in the private labor market. A reviewer of our manuscript proposed compellingly that veterans' preference might serve as a way that veterans identifying as Black or African American circumvent discrimination in the private labor market in order to obtain quality employment. In the second section of these supplementary materials, we consider that hypothesis using the data described above.

Basic questions about the policy implications of our findings also can be explored via the NARA-version of the CPDF. That is, the types of jobs that veterans staff, the authority they have to direct the work activities of other employees, and the agencies with which they are affiliated all can be studied by extracting simple summary statistics from the NARA-version of the CPDF. In these supplementary materials, we report analyses that do so.

2. Racial identities of employees receiving veterans' preference

2.1. Introduction. The effect of veterans' preference on the demographic composition of the U.S. federal government has received attention from both policy makers and researchers, with each acknowledging that veterans' preference largely benefits White males. For instance, before a Congressional subcommittee in 1977, Civil Service Commission Chairman Alan Campbell lamented that "preferential hiring from a labor pool which is 98% male and 92% nonminority places an inordinate burden on Federal agencies trying to implement affirmative action," (*3*, p.8; also quoted in 4). Writing in an academic journal article nearly four decades later, Gregory B. Lewis echoed Campbell's sentiments: "By explicitly preferring a group that has traditionally been very disproportionately male, White, native born, and heterosexual, veterans' preference creates obstacles to the diversity and may decrease the representativeness of the federal civil service" (*1*, p.247). However, a reviewer of our manuscript also provided the compelling hypothesis that veterans' preference might serve as a pathway for individuals identifying as Black to avoid discrimination, thus resulting in greater entry into U.S. federal employment among veterans identifying as Black. Such sentiments, and the reasoning behind them, lead us to examine the relationship between race and veterans' preference among the birth cohorts that we study in the main text.

In the subsequent sections, we explain our methods and results in detail, but here we provide a preview of those findings:

- First, we report that, as one would expect, given the composition of the armed forces and U.S. demographics at the time, individuals identifying as White constituted the largest portion of veterans' preference recipients among the draft-eligible cohorts that we study. This observation amounts to a validity check that ensures our results rest on a common foundation with intuition and past research.
- Second, the number of White males in federal employment steeply declined from draft-eligible birth cohorts with numbers called for induction to draft-eligible cohorts whose numbers were not called for induction, whereas the opposite trend occurred among males identifying as Black. Thus, our results suggest that the call of lottery numbers, on average, increased the number of White males in federal employment and decreased the number of Black males in federal employment, while leaving the number of Black women and White women unchanged.
- Third, with those results noted, and consistent with other past research (*1*, *4*), we also find that the majority of individuals identifying as Black or African-American in the study's birth cohorts entered with veterans' preference benefits. Thus, although veterans' preference predominantly benefits individuals identifying as White, it also appears to provide an avenue for individuals identifying as Black or African American to find quality employment, though this effect, as we explain, actually may have been dampened because of the call of lottery numbers in the VSSL (or another coincident phenomenon that affected the 1950-1952 birth cohorts).

These findings, which result from simple analyses of population data, provided a complex picture of the way that race and veterans' preference shape the ranks of the U.S. federal executive branch. In the following paragraphs, we elaborate on them.

2.2. Methods. To uncover the aforementioned patterns, we examined a publicly-available version of the CPDF stored by NARA spanning the years 1973-1997. The data consist of an annual cross-section of the CPDF taken in September. Each row of the data provides information concerning the professional, personal, and workplace

characteristics of a given employee—for instance, an employee's work location, pay, occupation, agency of employment, supervisory status, racial identity, sex, year of birth, month of birth, and veterans' preference status, not to mention a host of other salient pieces of information. Employees are identified by a unique identifier (a scrambled Social Security Number), which persists across the years of the data set. The data do not contain sensitive information such as an employee's home address or day of birth.

The exclusion of day of birth, plus the years these data cover, make them different from the version of the CPDF studied in the main text. As mentioned in the main text, the data studied therein cover only recent years when OPM officials first started storing day of birth information in the CPDF, whereas the version of the CPDF studied here covers a larger number of earlier years when OPM did not record day of birth (namely, 1973-1997). Also, the data in the main text do not include the Department of Defense, which moved, in recent years, to an independent personnel records system that separately stores its electronic employee data. Thus, we perform our analysis in these supplementary materials with both the Department of Defense included in our analysis (this section) and excluded from our analysis (Section 6 of these supplementary materials). Setting aside these differences, both the main text and these supplementary materials study a variant of the CPDF. Or, put differently, both sections of the manuscript focus on the exhaustive population of personnel records collected by OPM, though they differ in the above subtleties.

To execute our analysis, we subset the data to include birth cohorts previously studied in research using the VSSL—that is, those from 1950 to 1956 (see *5*). One data set consists exclusively of males born in this time period and the other consists of both males and females. On each of these sets, we perform the following procedures.

Because we seek to understand whether individuals of varying racial identities gain federal government employment differentially due to veterans' preference, we subset the data solely to include individuals in their first year of federal employment. This sub-setting results from data operations in which we, first, sort the data by each employee's unique identifier and, then, within each value of the unique identifier, sort by the calendar year of the data. These steps organize the data such that each employee's observations are stored in the annual order in which they were generated; we subsequently create a counter within values of the unique identifier that denotes the employee's "year of service." Next, we throw out observations with a year of service greater than 1. Because our data start in 1973, all employees working in the federal government in that year have "1973" as their first year of service; this means that we must also throw out those observations. Furthermore, the CPDF did not contain information about an employee's racial identity prior to 1976, thus we subset the data solely to include observations reported in the CPDF after 1975. This limitation of our data raises the possibility that we might undercount the effect of veterans' preference if individuals involved in the Vietnam conflict entered employment immediately upon return; if true, then the most pronounced undercount would be for the birth cohorts eligible for the first three lotteries. In total, these procedures reduce the data consisting solely of males to 953,287 observations, whereas it reduces the data set consisting of males and females to 1,499,705 observations. These observations include employees, born from 1950 to 1956, in their first year of federal employment, which took place after 1975. Finally, due to this section's focus on the relationship between employees' racial identities and whether they received veterans' preference, our analysis centers on two CPDF variables related to those attributes.

OPM classified individuals' racial identities—during the years under study—into one of 16 racial or national origin categories. These classifications were stored in a variable labeled "Race or National Origin," which we use in our investigation. The coding

system used for this variable includes overlapping designations for individuals identifying as Asian or as a Pacific Islander, which forces us to group individuals who hold such identities. For instance, it provides a category in which individuals can identify as “having origins in any of the original people of China” and live in Hawaii, as well as a category for individuals “who are Part Hawaiian and identify most closely with the Hawaiian category” (*9*, p.375). Due to this overlap, we group those codes into a single variable that denotes whether an individual identifies with one of several Asian national or cultural groups; this decision clearly masks heterogeneity in individuals’ identities, but we deem it the only method of providing consistency in our reporting in the context of OPM’s overlapping coding scheme.

Also, during the time period of our data, veterans’ preference was awarded via a point system in which the numerical score assigned to a job candidate’s application/entry exam (ranging from 0-100) was summed with either 5-points or 10-points depending on the level of preference benefits awarded to the applicant. Although the stipulations surrounding the receipt of 5- or 10-point preference are myriad, the primary consideration for 5-point is that a veteran served 180-consecutive days of active duty during a designated period of armed conflict and10-point preference is awarded for experiencing a service-related event that affected a veterans’ abilities. A very small percent of 10-point recipients consist of the family members of veterans who suffered injury or death during service. OPM created a variable “Veterans’ Preference” storing information that indicated the level of preference awarded. We created a binary indicator that denotes, via a value of unity, if an employee received any level of preference (5- or 10-point preference) and denotes those not receiving preference with a value of zero.

With these data, we first derive some simple summary statistics using the set consisting solely of males. In particular, we examine the percent of veterans’ preference recipients who identify with each of the racial or national groups that OPM uses in its classification scheme. Next, we examine the percent of each identity group’s male members who enter via veterans’ preference. Then, we perform a difference-in-difference-in-difference analysis that measures the effect of the VSSL call of lottery numbers on cohorts of Black male employees versus White male employees. After this analysis, we perform another such analysis examining the effect of the VSSL call on Black male employees, versus White male employees, who entered via veterans’ preference. Then, to gauge the robustness of our results further, we repeat our difference-in-difference-in-difference analysis solely with the 1952 and 1953 cohorts, thus making a comparison across adjacent birth cohorts that one would expect to exhibit greater similarity.

2.3. Results. Among male employees eligible for the VSSL, we discover patterns similar to previous research (*1, 4*). Past research finds that the vast majority of veterans’ preference recipients are White, but, among various racial or national identity groups, the majority of individuals identifying with those groups are veterans’ preference recipients (*1, 4*). Table S1, which appears on page S21, shows the percent distribution of male employees’ racial identities, by birth cohorts in the VSSL, separated by whether or not they entered with veterans’ preference. Across all birth cohorts, over 60% of the male employees entering with veterans’ preference are White. However, the meaning behind this figure becomes obscured once one notes that, among non-recipients of veterans’ preference, an even greater percent of male employees identify as White. In other words, veterans’ preference largely benefits White employees, *but* those employees already constituted the largest portion of the federal executive branch (see also *4* for this interpretation). In contrast, individuals identifying as Black comprised between 20% and 27% of all employees entering with veterans’ preference across the VSSL birth cohorts, yet individuals identifying as Black comprised only about 9% to 11%

of the pool of male employees entering without veterans' preference benefits. A similar pattern appears among individuals identifying as Hispanic.

To understand this pattern more clearly, we switch the denominator from the column margin of the underlying table of counts to the row margin, thus examining the proportion of employees identifying with a given racial or national origin category who received / did not receive veterans' preference benefits (Table S2, p.S22). Table S2 more clearly shows that, across several groups, a greater proportion of the groups' members enter with veterans' preference benefits than without them. This pattern coincides with the hypothesis that members of those groups use veterans' preference as a means to secure federal employment. Moreover, the same pattern does not appear among White male employees—again, a finding consistent with the hypothesis that veterans' preference makes a notable difference in the pursuit of employment for individuals who identify with groups subject to discrimination in the private labor market, but not to members of groups that do not suffer from discrimination in the labor market.

However, albeit consistent with that hypothesis, other interpretations are possible. It could be that participation in the military changes preferences for public employment differentially across individuals' of varying racial identity groups, thus leading members of some groups to gravitate to public employment at greater rates. It, also, could be that the data fail to account for attributes of military service—say, military occupations—that co-vary with both racial identities and the attributes of federal employment—again, say, the occupations in the civilian federal government. In sum, selection bias and confounding factors could undermine the interpretation of the evidence reported here and, thus, caution is warranted in any effort to explain why these patterns appear.

The above summary statistics offer broad insight into the relationships between veterans' preference and racial identities, but how did the call of numbers in the VSSL affect these relationships? Ideally, to address this question, we would replicate the research design reported in the main text on subsets of data consisting of groups of employees with differing racial identities. We lack such data, however, due to the sensitive nature of combining individuals' birth dates with information about their racial identities. In lieu of using that research design, we compare how the average number of employees identifying as, respectively, Black females, Black males, White females, and White males changes from birth cohorts called for induction (1950-1952) to those not called. This analysis amounts to a difference-in-difference-in-difference (DDD) design.

Figure S1 (p.S11) reports the key takeaway from this analysis. The average number of employees identifying as Black males, Black females, or White females grew slightly from the birth cohorts called for induction (1950-1952) to the birth cohorts not called for induction (1953-1956), yet the average declined among White males from one birth cohort to the next. Please note that, given our investigation of the population, this change cannot result from chance variation in sampling (i.e. it requires no inferential statistics to establish it). In Table S3 (p.S23), results from a regression implementation of this DDD indicate that birth cohorts of White males called for induction had 16,609 employees more than the White male birth cohorts not called for induction, conditional on the separate effects of sex, race, the induction call, and their interactions.

To explore how the above patterns related to veterans' preference, we examined the mean number of employees across the above, racial identity groups when also taking into account veterans' preference status. Figure S2 (p.S12) reports these results and shows a steep decline in the average number of White-male veterans' preference recipients from the birth cohorts called for induction (1950-1952) to the draft-eligible, White-male cohorts not called for induction (1953-1956). These figures correspond to what one would expect if lottery numbers called for induction affected the ranks of the

federal service and did so by increasing the number of White male veterans. Noticeably, the average number of veterans' preference recipients identifying as Black males also declined from the 1950-1952 birth cohorts to the 1953-1956 birth cohorts, thus suggesting that the VSSL modestly increased the number of Black males who could enter the federal service via veterans' preference. Consistent with the eligibility criteria of the VSSL and military policies of the time, a very small number of females entered with veterans' preference at the time and their levels did not change in relation to the VSSL. Finally, all groups of employees not receiving preference appear to have increased their numbers from the birth cohorts called for induction (1950-1952) to the cohorts not called (1953-1956).

Regression analyses appearing in Table S4 (p.S24) provide information about the magnitude of the changes across birth cohorts shown in Figure S2. The regression analyses suggest that the VSSL call for induction added approximately 10,834 White-male veterans' preference recipients to the federal government, conditional on the effects of cohort sex, race, status as a 1950-1952 birth cohort, receipt of veterans' preference, and the interactions of those variables.

The preceding analyses result from the aggregation of cohorts called for induction and those not called for induction, which led us to wonder whether differences in the sheer volume of military veteran employees across cohorts might explain the results. We also worried that averaging across birth cohorts might create anomalies that could influence results. To address these problems, we repeated the analyses that appear in Figures S1 and S2 using only employees born in 1952 and 1953. Not only are these birth cohorts temporally adjacent, but, also, they exhibit greater similarity in the number of employees in each cohort, thus providing a way to reduce the possibility that variation in the volume of veterans and employees across cohorts underlies the effect we observe. Plus, by studying only two cohorts, we need not take averages across birth cohorts. As Figure S3 (p.S13) indicates, we find a pattern in these two birth cohorts that is similar to that observed in Figure S1, which involves all of the birth cohorts under study. Specifically, among the identity groups being studied, we only find a prominent decrease in the number of White males when examining employment levels between the birth cohort with numbers called for induction (1952) and the birth cohort that did not have numbers called for induction (1953). This finding further suggests that the VSSL's effect on federal employment passed primarily through its effect on the number of White male employees. Figure S4 (p.S14) provides further evidence to that effect; it shows that only the number of White, male veterans' preference recipients appeared to decline from the 1952 birth cohort to the 1953 birth cohort. No other identity group being studied shows a similar pattern, thus suggesting that the draft lottery's effect on employment in the federal service appears to have come primarily through increased employment of White male veterans.

2.4. Conclusion. These results coincide with the observations starting this section: veterans' preference appears to add White males to the federal service, though individuals identifying as Black males often enter service benefitting from it. The results also suggest that the VSSL's induction call magnified the effect of veterans' preference. These findings carry important policy implications. Previous research shows that the nature of how and what public servants deliver in policy outputs varies according to the degree to which those public servants reflect the identities of their clientele (*6*). Thus, if the VSSL call for induction led, via veterans' preference, to the federal civil service becoming more White and male, then one would expect the demographic representation of the federal service to differ from that of the public writ large and this could influence policy outputs. Future research should consider this possibility as it explores, in detail, the policy implications of the phenomenon we report here.

3. Attributes of jobs staffed by veterans' preference recipients upon entry

Previous research indicates that the distribution of veterans' preference recipients across agencies, occupations, and other workplace characteristics differs from that of employees who do not benefit from veterans' preference (*7*). In this section, we examine whether similar differences exist within the male birth cohorts studied in the main text.

Examining these differences allows us to speak to the policy implications of our findings. Although the influence of street-level bureaucrats over the determination of policy outputs is well-established (*8*), higher-level positions in the public bureaucracy have wider influence over broad, stated policies. Thus, an analysis of the positions that veterans' preference recipients fill lends insight into the type and magnitude of the policy influence that they might have. Furthermore, if veterans' preference recipients largely fill lower-level, street-level posts, then this finding also suggests that the most significant policy implications of our findings might relate to the labor market, the transition of veterans to civilian life, the representativeness of the public bureaucracy, and civil-military relations, not to the policy discretion and politics of the administrative state.

3.1. Occupational categories. The U.S. federal government places occupations into six categories: blue collar, administrative, clerical, professional, technical, and other white collar. Blue-collar occupations differ from the latter five categories in that they involve, even in supervisory positions, "the trades, crafts, and manual labor (unskilled, semiskilled, and skilled)" (*9*, p.305). The other occupations are so-called, "white-collar" occupations. Administrative and professional occupations are defined by the knowledge that the holder must possess. The latter require the ability to know and apply managerial know-how, whereas the former entail knowledge in an academic specialization (e.g., chemistry). Technical occupations are positions that assist personnel in administrative or professional roles, whereas clerical positions entail routinized operational tasks (e.g., office work, financial processing, and so on). Other white-collar occupations do not fit in these categories, yet also do not involve the types of manual labor or trade tasks found in blue collar positions. As Table S6 (p.S26) indicates, the recipients of veterans' preference disproportionately congregate in blue-collar positions and, relative to non-recipients of preference, in clerical occupations.

3.2. Supervisory status. The CPDF also denotes whether an employee is a supervisor of other's work. That is, whether the employee has managerial duties that involve directing and managing other employees. The vast majority of employees—regardless of veterans' preference status—do not have such authority, but we find slightly lower levels of supervisory status among veterans' preference recipients (see Table S7, p.S27).

3.3. Agency affiliations. Previous research notes that veterans' preference recipients populate some agencies more than others and, thus, we again examine in this section whether this previously observed pattern holds among the birth cohorts of draft-eligible employees. Figure S5 (p.S15) indicates that preference recipients in the draft-eligible cohorts largely congregate in agencies relating to the military or to veterans. Roughly 20% of all preference recipients, for instance, start their federal service in the Navy or in the Department of Veterans Affairs/Administration, while less than 5% work in the Department of Justice, the Department of Agriculture, or the Department of the Treasury. While a thorough investigation of this pattern extends beyond the scope of these supplementary materials, we speculate that familiarity with the occupations, agency structure, and culture of agencies related to the armed forces makes veterans' preference recipients more inclined to work in them.

4. Effect on the productivity or performance of the civilian U.S. federal government

Given the substantial portion of the civilian U.S. federal executive branch staffed by veterans' preference recipients, researchers in public administration have paid recent attention to whether these employees perform better, worse, or comparably to other employees. Consistent with the notion that veterans' preference allows employees to escape thorough scrutiny upon hire, initial evidence suggested that veterans reached higher grades in the federal General Schedule pay ladder—a widely used approximation for job performance—at a more drawn-out pace than nonveterans (*1*). However, subsequent research found that this difference in career advancement vanished once one controlled for the positions (that is, occupations, agencies, and duty stations) that preference recipients and non-recipients entered upon starting federal employment (*7*). More-recent research using employee pay as a proxy for performance also found comparable levels of performance across veterans and nonveterans (*2*).

In this section, we apply the research design used in these most-recent investigations (viz. *2*, *7*) to data solely concerning federal employees eligible for the VSSL. Specifically, we subset the overall CPDF to include males born from 1950-1956 and order them, as described in section 2.2 above, so that we can create a counter variable that denotes the employee's year of service. Given the time span of our data, we exclude observations whose first year was prior to 1974 (as we cannot verify the actual first year of employment of those employees) and we delete all observations for employees whose personnel identifier appeared twice in a given year of data (i.e. all observations associated with a duplicated ID). The latter step eliminated less than 1% of all observations and left us with 5,224,764 employee-year observations.

We, then, created our focal independent variable—a binary indicator that took a value of one if an employee received veterans' preference benefits and a zero if the employee did not. We also recorded the first agency, first duty station, first occupation, and first calendar year in which the employee entered federal service, as well as their first inflation-adjusted annual pay. All income in our analysis is inflation-adjusted to 2019 dollars. The variables in the CPDF used to measure pay are "salary" and, then, "basic pay" (salary was the base-level compensation variable in the CPDF prior to 1987). We use the logarithm of these variables, after the first year of employment, as the dependent variable in our investigation. Consistent with the past literature (*2*), we use pay as a proxy for the productivity/performance of an employee.

Variables concerning an employee's work situation in the first year of employment serve as a way to pair preference recipients with non-recipients entering employment in the same conditions. Doing so allows us to compare, across each year of service, the log pay of preference recipients against that of non-recipients who entered service in the same conditions, thus creating a counterfactual for whether the employees starting in those conditions would have advanced further (i.e. performed better) if they were preference recipients or non-recipients. We execute this comparison by taking the difference in the logarithm of inflation-adjusted annual pay between veterans' preference recipients and non-recipients, using the matching procedures described in *10*.

Consistent with ref. 2 and 10, we find little evidence of substantial differences in the performance of veterans' preference recipients and non-recipients, as measured by differences in inflation-adjusted pay. Table S7 (p.S27) presents these results by displaying the difference in the logarithm of inflation-adjusted pay, between preference recipients and non-recipients, across the first 10 years of their federal service, conditional on the matching variables denoted in the final five rows of each column. For instance, each value in the column labeled "Matching Combination 1" reports the average difference in the logarithm of inflation-adjusted pay for preference recipients and

non-recipients who entered federal employment in the same agency, duty station, occupation, and year, and with the same income. Due to the properties of logarithms, these estimates can be used to compute percent changes; when one does so, it becomes apparent that in none of the years of employment studied do preference recipients and non-recipients differ by more than 1% in their log inflation-adjusted pay. We interpret this evidence as effectively indicating no marked difference in productivity.

5. Position attributes and productivity by veterans' preference status and race

In the previous sections of these supplementary materials, we have examined the relationship between (i) race and veterans' preference status, (ii) veterans' preference status and the positions individuals work in the federal executive branch, and (iii) the relationship between veterans' preference status and productivity. This section explores the intersection of these relationships by presenting evidence that lets us assess whether the positions and productivity of veterans' preference recipients vary by the race with which they identify.

As Tables S8 – S10 (pp.S28-S30) indicate, we find small differences in how veterans' preference recipients identifying as Black, versus veterans' preference recipients identifying as White, allocate themselves across positions or advance to higher levels of pay (i.e. our proxy for productivity). Veterans' preference recipients identifying as either Black or White work in blue-collar positions at higher rates than non-recipients of their same identity group (see Table S8, p.S28). Preference recipients identifying as White exhibit a greater likelihood of filling an administrative or professional position—compared to their non-recipient peers—than preference recipients identifying as Black do in comparison to their non-recipient peers who share the same identity (see Table S8, p.S28). In terms of supervisory status, veterans' preference recipients identifying as Black are, across birth cohorts, only several percentage points less likely to serve in a supervisory role than non-recipients sharing the same racial identity, whereas White preference recipients are about twice that many percentage points less likely to perform in a supervisory role than White non-recipients (Table S9, p.S29). Finally, in regards to productivity, preference recipients identifying as Black or White differ in their pay from non-recipients of the same identity by less than one percent in absolute terms. Thus, if pay serves as a reliable proxy for productivity, then these findings suggest that not only do minor differences exist between the productivity of preference recipients and that of non-recipients, but this minor difference does not vary by the racial identity of the employees being studied (Table S10, p.S30). In sum, the differences between preference recipients and non-recipients appear relatively uniform across groups of employees identifying as Black or White.

6. Replication of analyses in sections 1-4 excluding the Department of Defense

Data in the main text of our manuscript do not include employees from the Department of Defense because that agency moved to its own unique personnel records system in recent years. However, as mentioned in Section 2 of these supplementary materials, the data we study in the supplementary materials comes from an era in which the Department of Defense reported its electronic personnel records to the Office of Personnel Management; thus, analyses in these supplementary materials, thus far, have included the Department of Defense. We believe that approach is justified on the principles that drawing on the entirety of the available data maximizes the generality of our findings. However, to ensure parity with the analysis in the main text, these

supplementary materials also report analyses in which we exclude the Department of Defense. Ultimately, our findings indicate that results differ little whether one includes or excludes the Department of Defense.

We present those findings in the same sequence as our results that include the Department of Defense (i.e. Table S1 [which includes the Department of Defense] compares with Table S11 [which excludes the Department of Defense], Table S2 compares with Table S12, Table S2 with S13, and so on). We begin with an analysis of the racial identities of individuals receiving veterans' preference, including how the draft lotteries altered the number of individual identifying with various identity categories. Subsequently, we examined attributes of the positions in which veterans' preference recipients work (e.g., agency affiliation, occupational category, and so on). Then, we consider differences in the productivity of preference recipients and non-recipients. Together, these analyses replicate the prior sections of these supplementary materials with employees of the Department of Defense excluded from the data. The results appear in Tables S11 – S20 (pp.S31-S50) and Figures S6 – S10 (pp.S16-S20), and they indicate that the exclusion of the Department of Defense does not alter the substantive interpretation of our findings.

Figure S1.
Mean number of employees by identity group and birth cohort

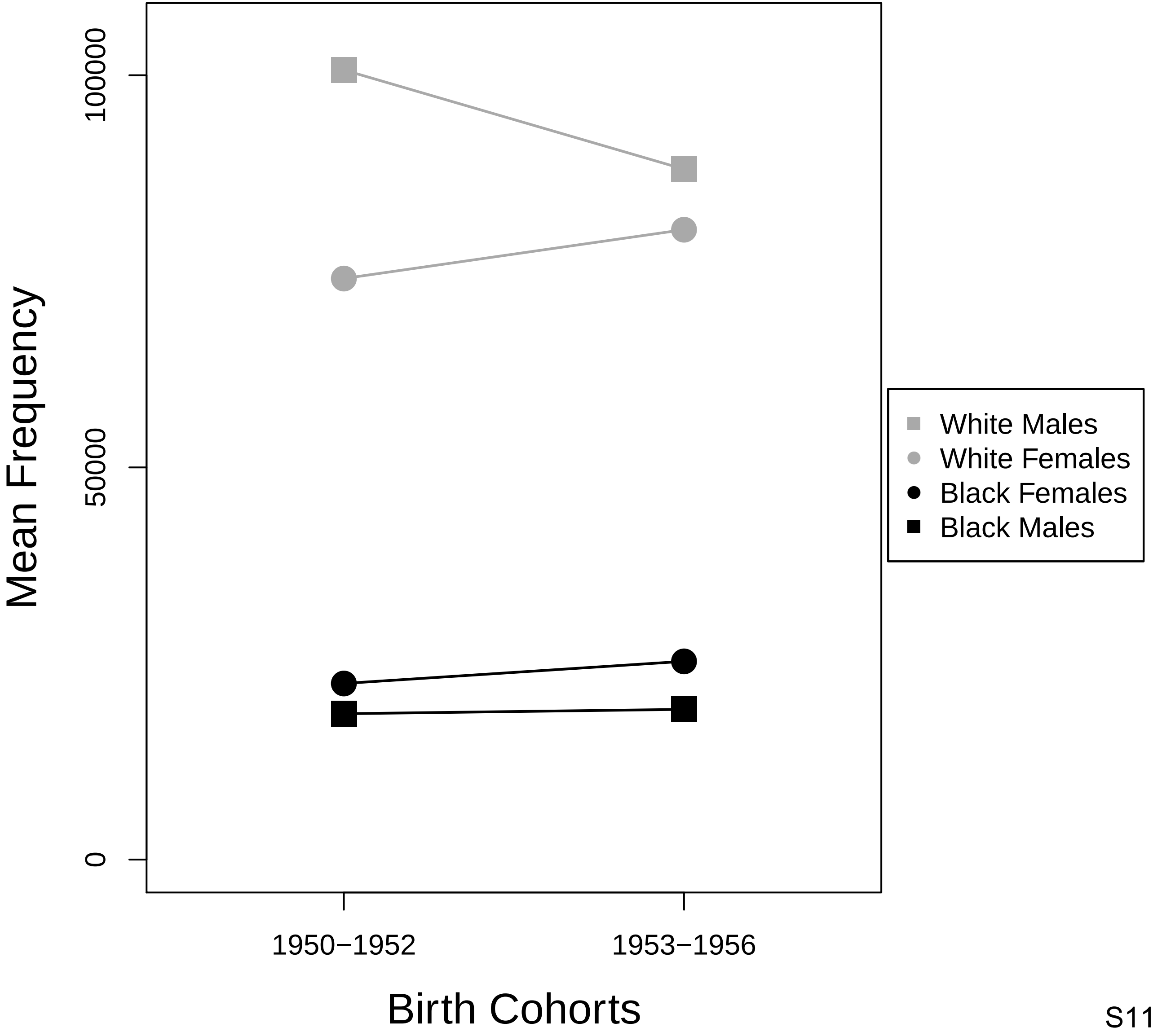

Figure S2.
Mean number of employees by identity group, birth cohort, and veterans' preference status

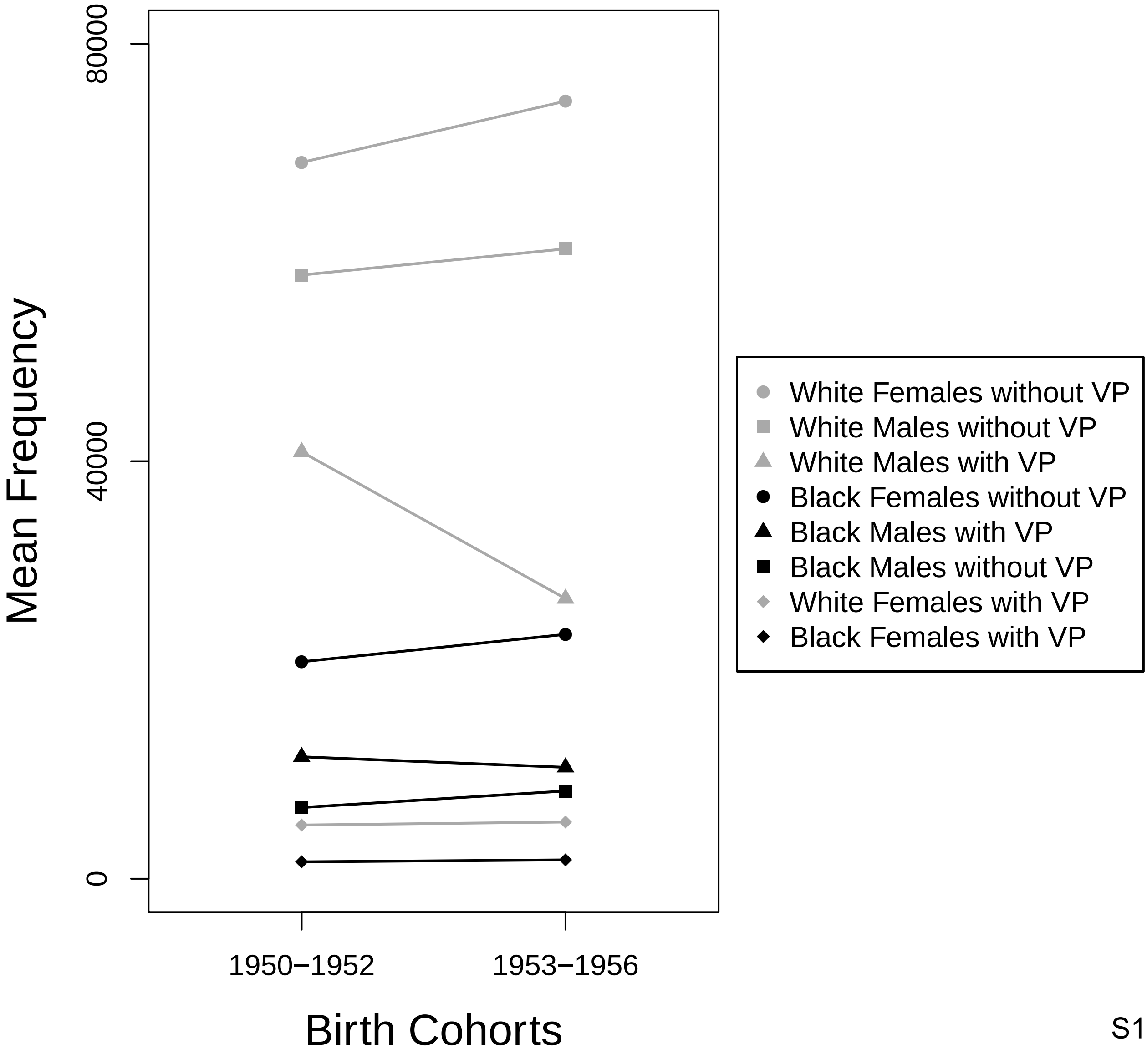

Figure S3.
Mean number of employees by identity group and 1952/1953 birth cohort

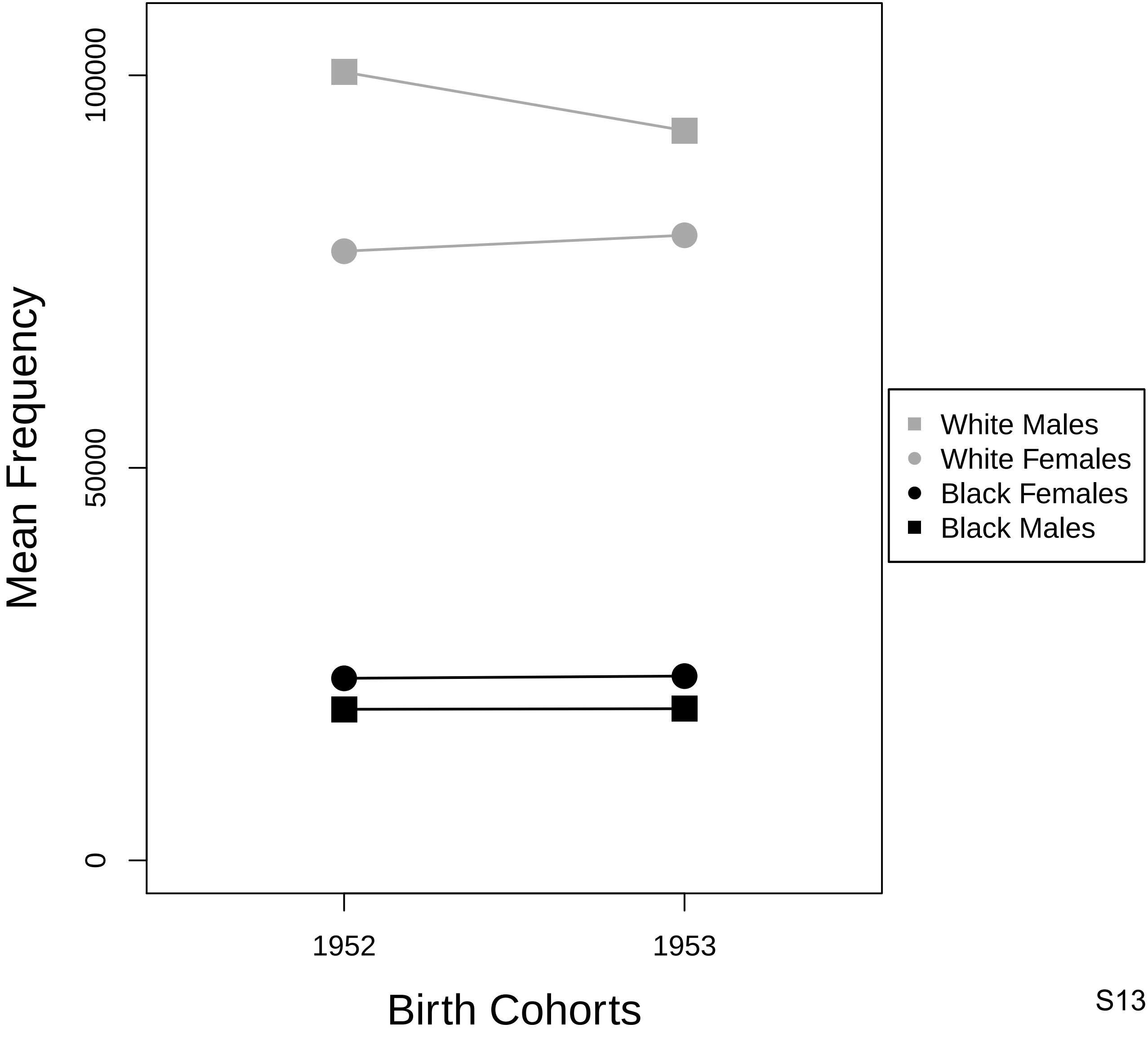

Figure S4.
Mean number of employees by identity group, 1952/1953 birth cohort, and veterans' preference status

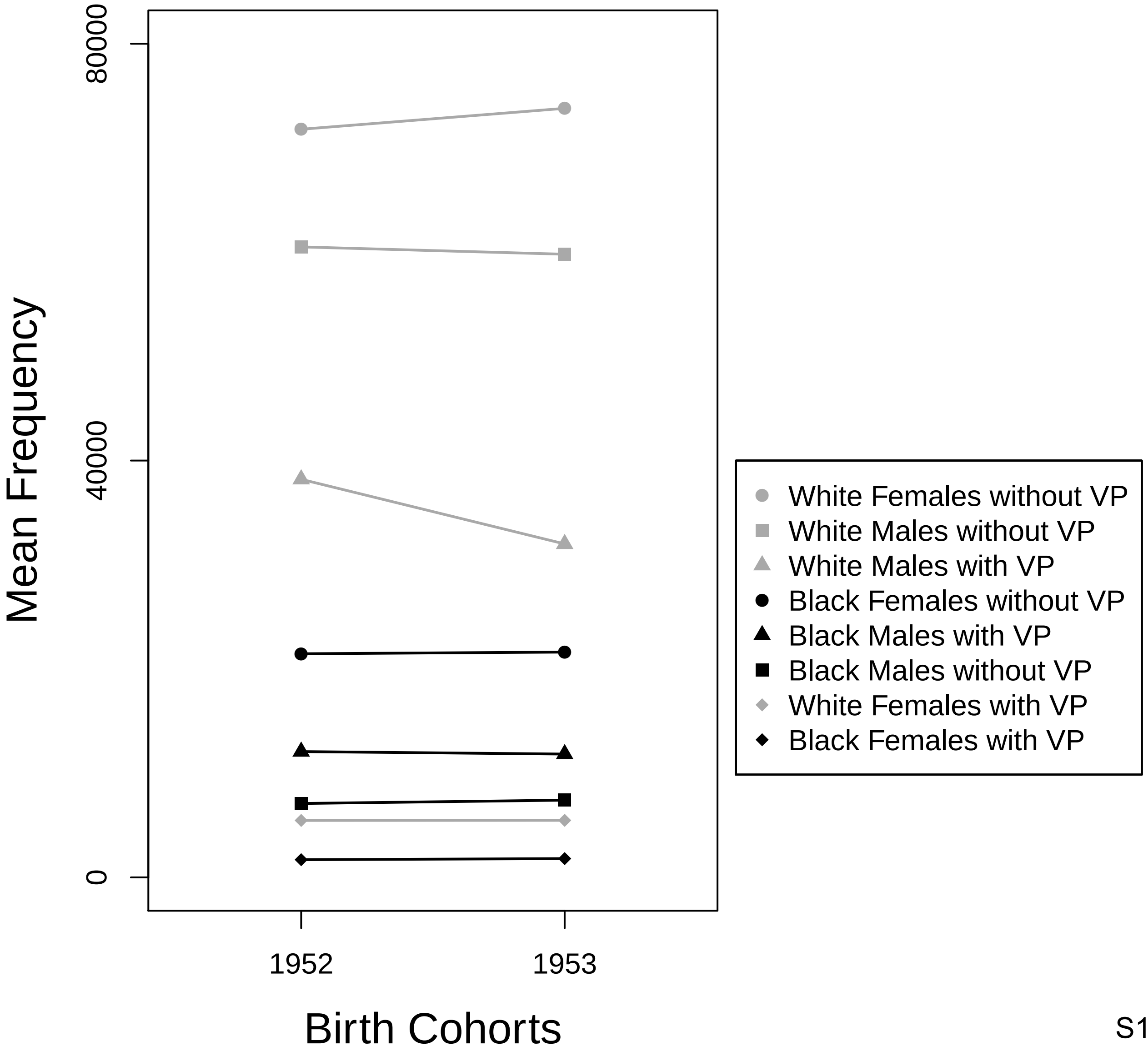

Figure S5. Proportion of Veterans' Preference Recipients across Agencies

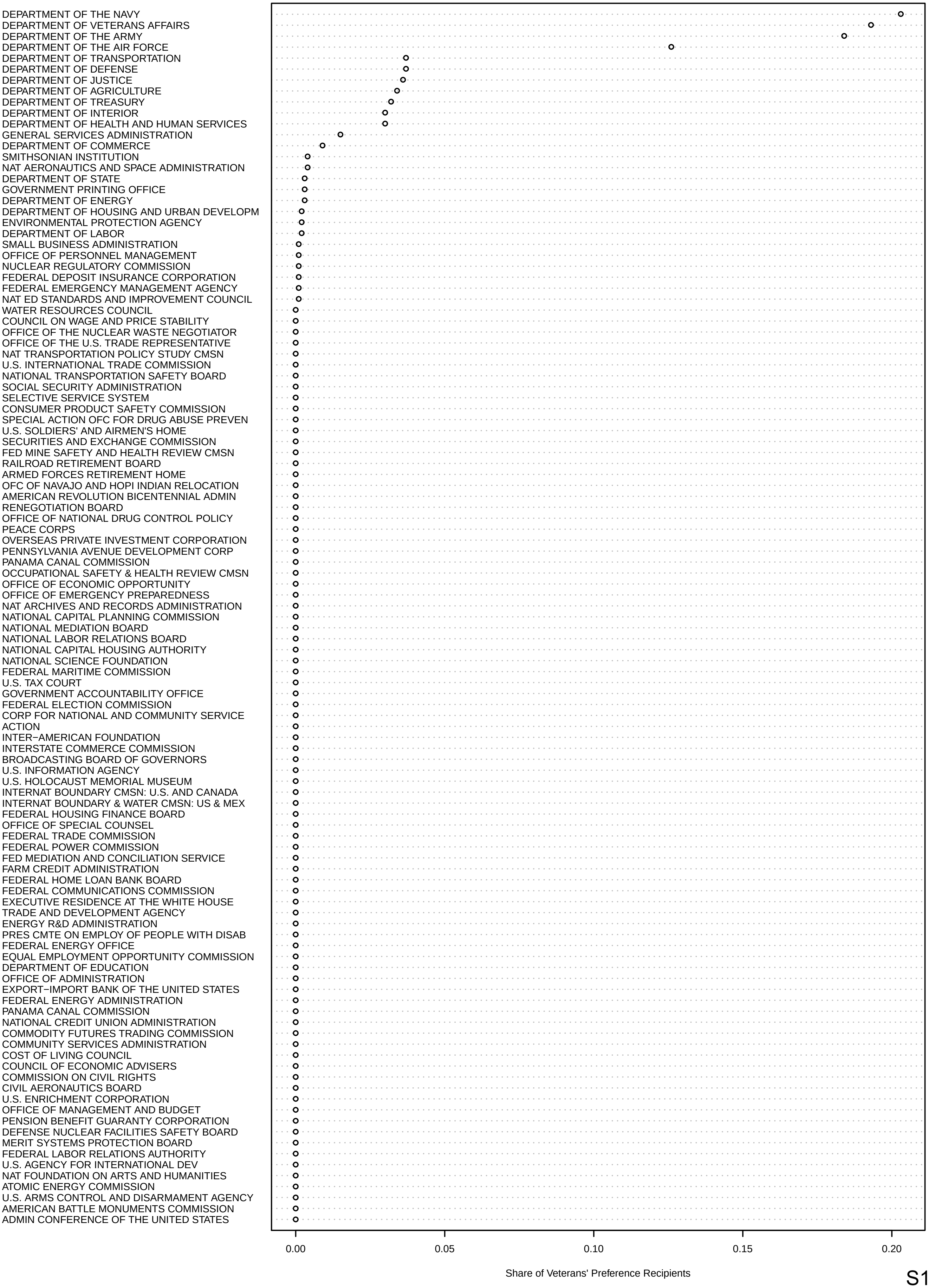

Figure S6.
Mean number of employees by identity group and birth cohort (No DoD)

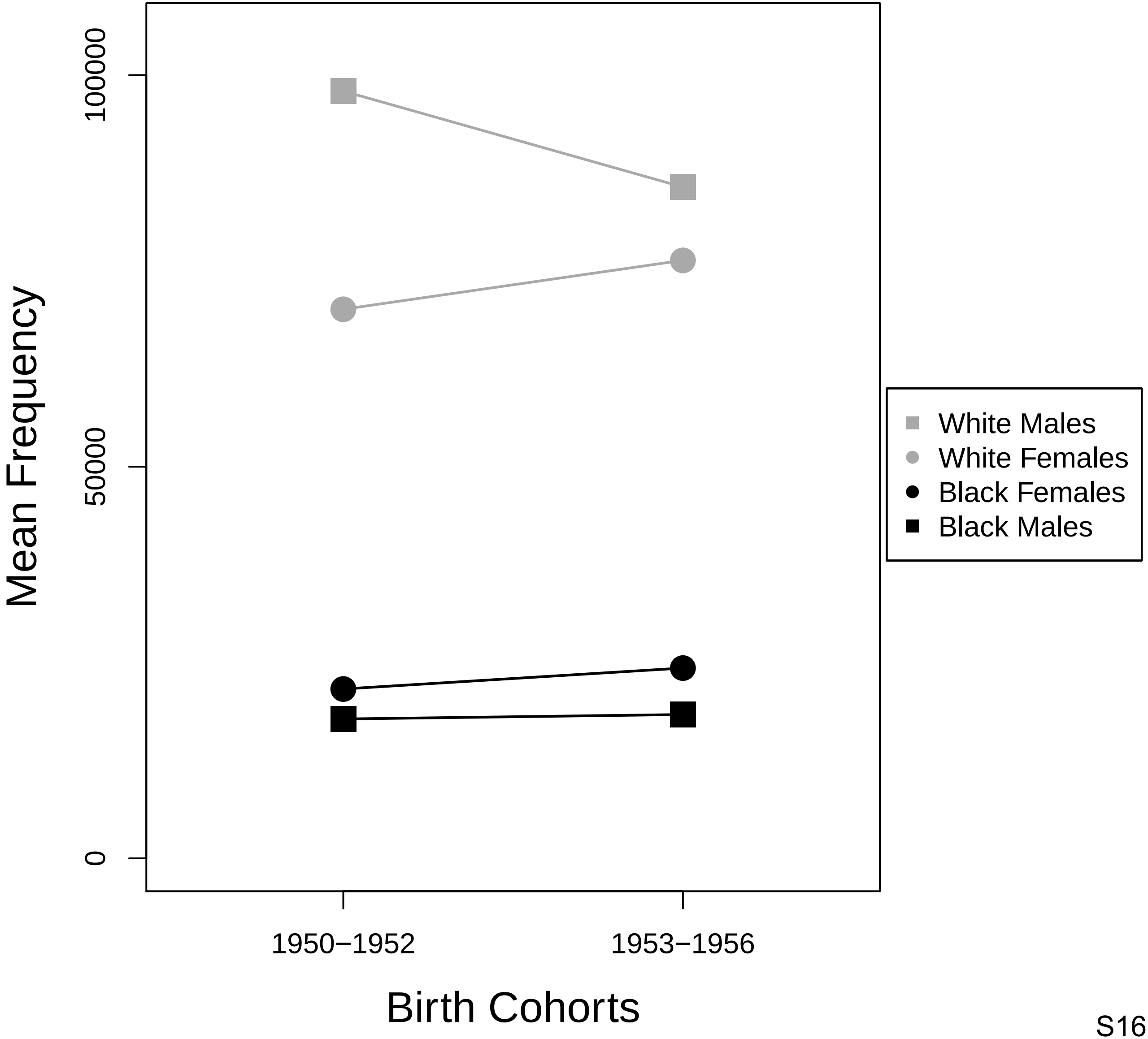

Figure S7.
Mean number of employees by identity group, birth cohort, and veterans' preference status (No DoD)

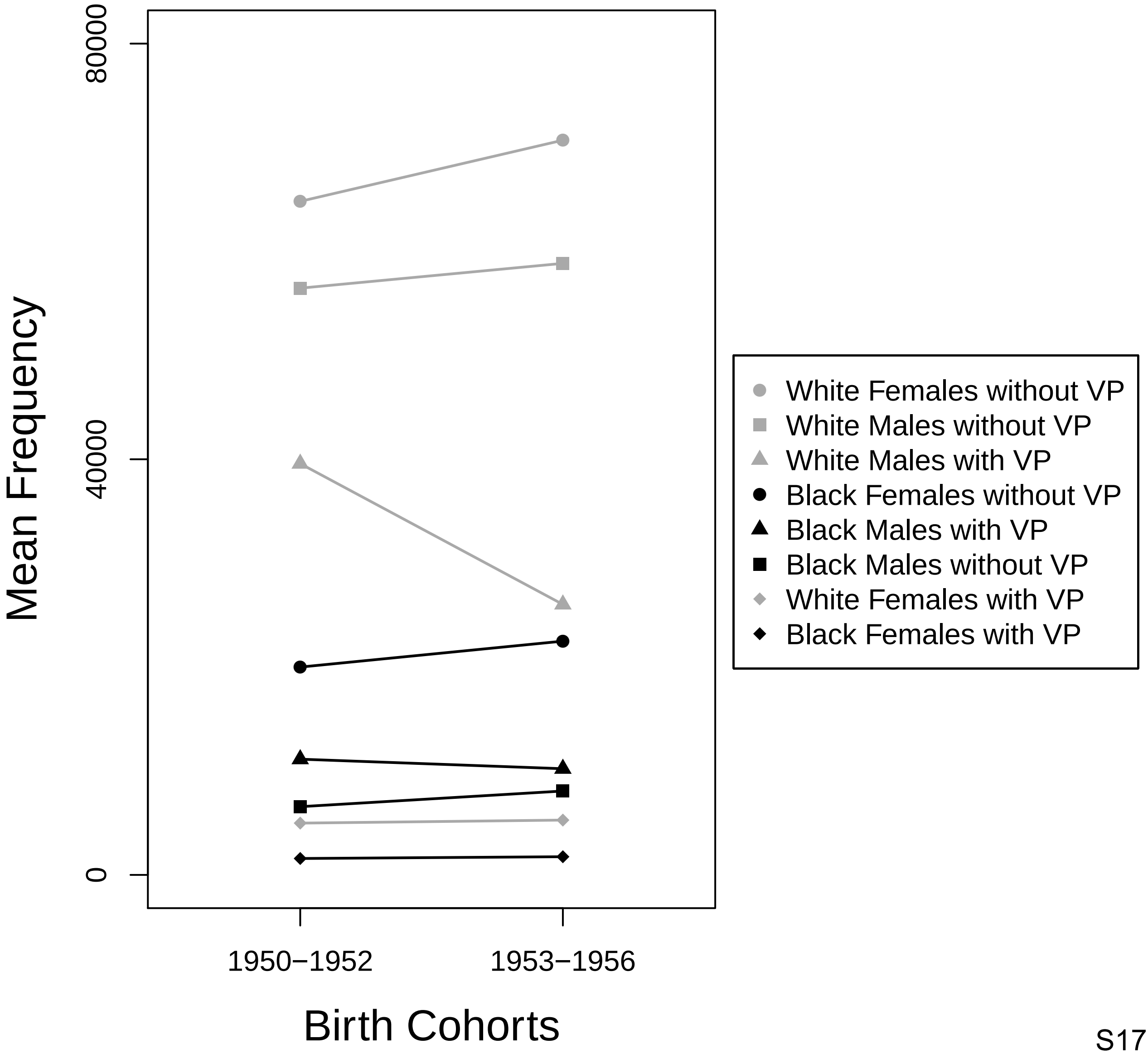

Figure S8.
Mean number of employees by identity group and 1952/1953 birth cohort (No DoD)

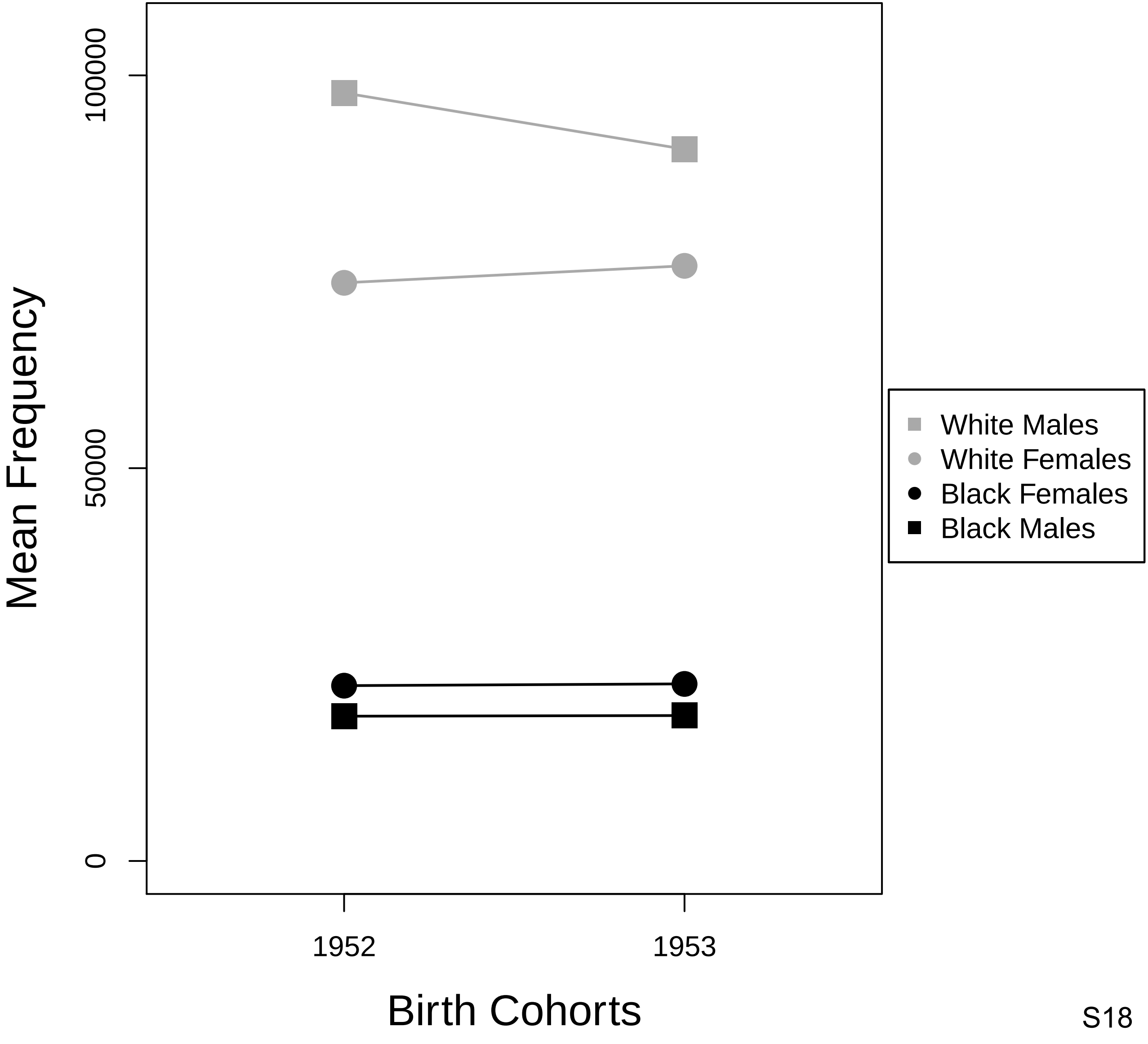

Figure S9.
Mean number of employees by identity group, 1952/1953 birth cohort, and veterans' preference status (No DoD)

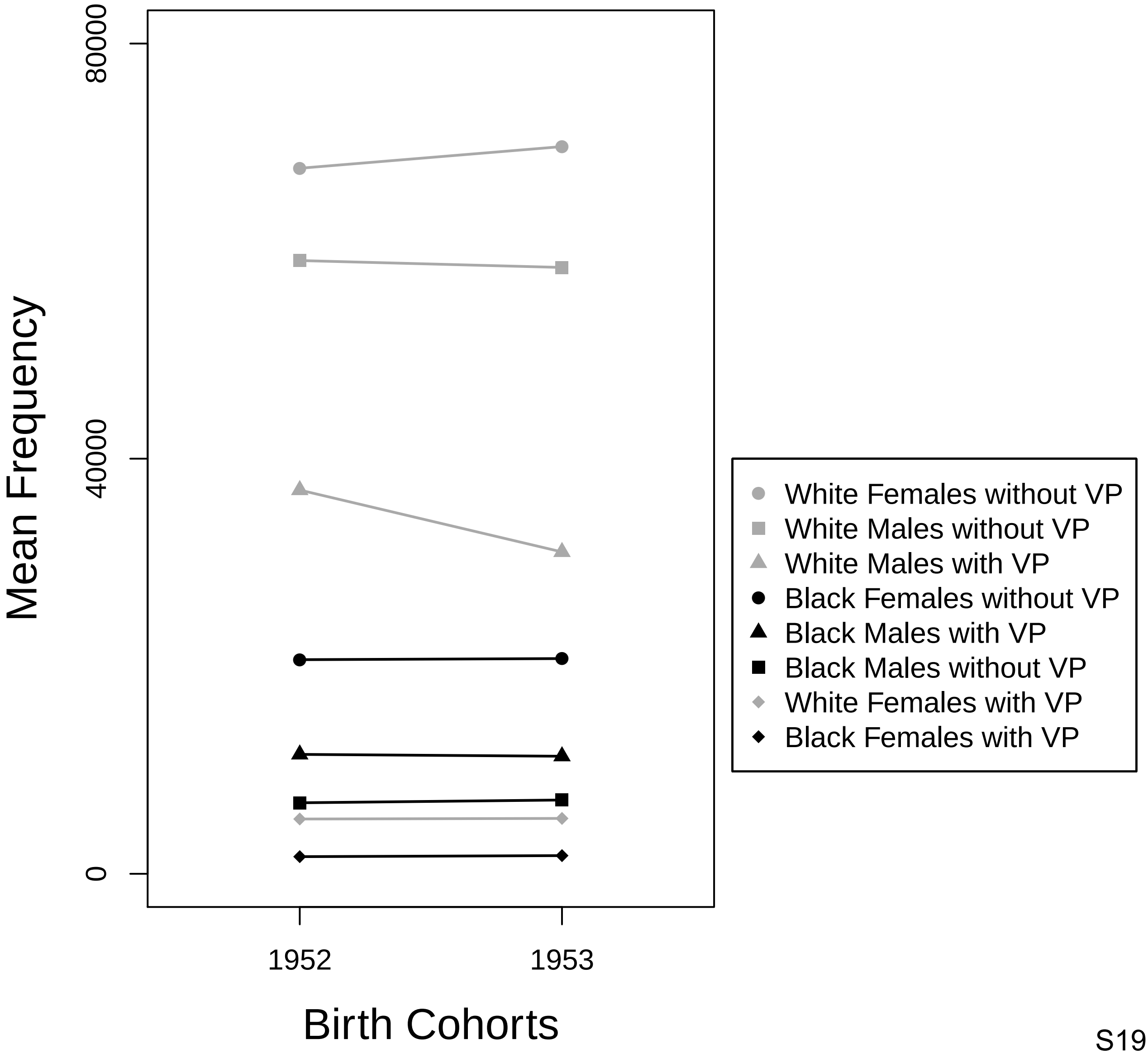

Figure S10.
Proportion of Veterans' Preference Recipients across Agencies (No DoD)

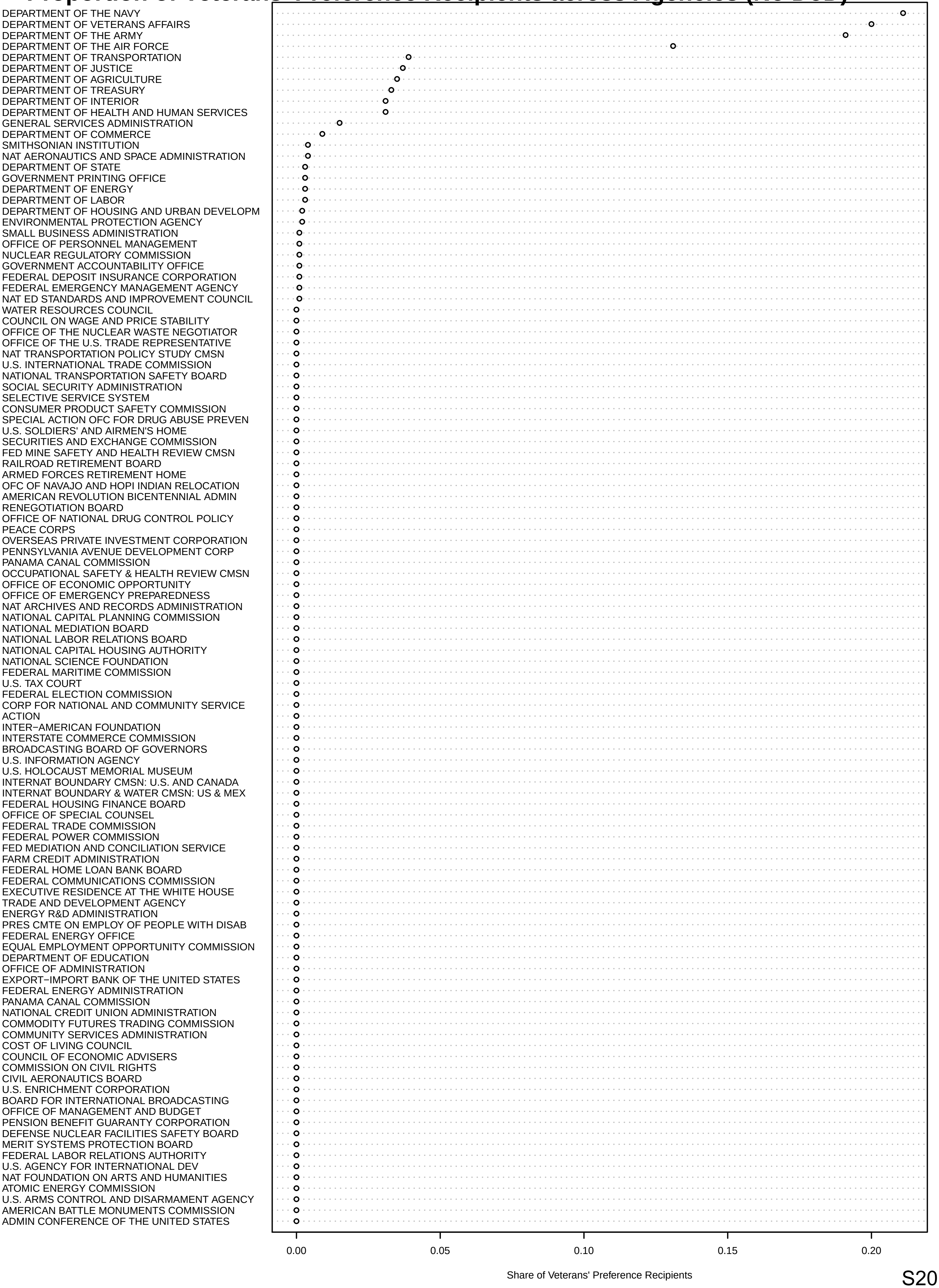

Table S1.
Proportion of non-Recipients and recipients of veterans' preference by identity group

	1950 Birth Cohort		1951 Birth Cohort		1952 Birth Cohort		1953 Birth Cohort		1954 Birth Cohort		1955 Birth Cohort		1956 Birth Cohort	
Racial or National Identity Group	No VP	VP	No VP	VP	No VP	VP	No VP	VP	No VP	VP	No VP	VP	No VP	VP
American Indian or Alaskan Native	0.02	0.02	0.02	0.01	0.02	0.01	0.02	0.01	0.02	0.01	0.02	0.01	0.02	0.01
Asian or Pacific Islander	0.04	0.03	0.04	0.03	0.04	0.03	0.04	0.03	0.04	0.03	0.04	0.02	0.04	0.02
Black, not of Hispanic Origin	0.09	0.18	0.1	0.2	0.09	0.21	0.09	0.24	0.1	0.25	0.11	0.27	0.11	0.25
Hispanic	0.04	0.07	0.04	0.08	0.04	0.07	0.05	0.08	0.05	0.08	0.05	0.08	0.05	0.08
Not Hispanic in Puerto Rico	≈0	≈0	≈0	≈0	≈0	≈0	≈0	≈0	≈0	≈0	≈0	≈0	≈0	≈0
White, not of Hispanic Origin	0.8	0.71	0.8	0.68	0.81	0.67	0.8	0.64	0.79	0.63	0.79	0.61	0.78	0.64

Note. The table displays, for each birth cohort in the VSSL, the proportion of veterans' preference non-recipients and recipients, respectively, identifying with each of the racial or national identity groups listed in the left hand margin of the table. The proportions of non-recipients across identity groups, in each birth cohort, appears under the column heading "No VP." The proportions of recipients across identity groups, in each birth cohort, appears under the column heading "VP." Thus, the denominator for each of the decimal values in, say, the column labeled "No VP," under the heading "1950 Birth Cohort," is the total number of employees in the 1950 birth cohort who did not receive veterans' preference benefits and the numerator is the number of said individuals identifying with the racial or national identity group listed in the corresponding left-hand margin. Values are rounded.

Table S2.
Proportion of identity group by receipt of veterans' preference

	1950 Birth Cohort		1951 Birth Cohort		1952 Birth Cohort		1953 Birth Cohort		1954 Birth Cohort		1955 Birth Cohort		1956 Birth Cohort	
Racial or National Identity Group	No VP	VP	No VP	VP	No VP	VP	No VP	VP	No VP	VP	No VP	VP	No VP	VP
American Indian or Alaskan Native	0.55	0.45	0.64	0.36	0.63	0.37	0.68	0.32	0.72	0.28	0.75	0.25	0.8	0.2
Asian or Pacific Islander	0.59	0.41	0.62	0.38	0.61	0.39	0.65	0.35	0.7	0.3	0.74	0.26	0.79	0.21
Black, not of Hispanic Origin	0.34	0.66	0.38	0.62	0.36	0.64	0.37	0.63	0.39	0.61	0.42	0.58	0.52	0.48
Hispanic, not identifying as White	0.38	0.62	0.41	0.59	0.42	0.58	0.47	0.53	0.49	0.51	0.52	0.48	0.6	0.4
Not Hispanic in Puerto Rico	0.59	0.41	0.69	0.31	0.68	0.32	0.77	0.23	0.75	0.25	0.71	0.29	0.69	0.31
White, not of Hispanic Origin	0.53	0.47	0.6	0.4	0.6	0.4	0.64	0.36	0.67	0.33	0.7	0.3	0.74	0.26

Note. The table displays, for each birth cohort in the VSSL, the proportion of each racial or national identity group that did not receive and did receive veterans' preference benefits. The proportions of non-recipients across identity groups, in each birth cohort, appears under the column heading "No VP." The proportions of recipients across identity groups, in each birth cohort, appears under the column heading "VP." Thus, the denominator for each of the decimal values in, say, the column labeled "No VP," under the heading "1950 Birth Cohort," in the row labeled "White, Not of Hispanic Origin" is the total number of employees in the 1950 birth cohort who identified as White, Not Hispanic, and the numerator is the number of said individuals who did not receive veterans' preference. Values are rounded.

Table S3.
Estimates from Difference-in-Difference-in-Difference Model

Parameter	Estimate
Intercept	25274<br>(1164)
Cohort identifies as male (0, 1)	-6124<br>(1646)
Cohort identifies as White (0, 1)	55037<br>(1646)
Cohort born from 1950-52 (0, 1)	-2821<br>(1778)
Cohort identifies as male (0, 1) *x Cohort identifies as White* (0, 1)	13838<br>(2328)
Cohort identifies as male (0, 1) *x Cohort born from 1950-52* (0, 1)	2266<br>(2515)
Cohort identifies as White (0, 1) *x Cohort born from 1950-52* (0, 1)	-3406<br>(2515)
Cohort identifies as male (0, 1) *x Cohort identifies as White* (0, 1) *x Cohort born from 1950-52* (0, 1)	16609<br>(3556)
R^2	0.9964
N (Unit: Birth Cohort-Gender-Race Grouping)	28

Note. Estimates stem from the regression of number of employees in birth cohort t, Y_t, on the variables and interactions appearing in the left hand column. Data come from the population, thus standard errors serve as a metric of variability across annual birth cohorts and do not offer a measure of sampling variation. Values are rounded.

Table S4.
Estimates from Difference-in-Difference-in-Difference-in-Difference Model

Parameter	Estimate
Intercept	23417<br>(1058)
Cohort identifies as male (0, 1)	-15011<br>(1497)
Cohort identifies as White (0, 1)	51087<br>(1497)
Cohort born from 1950-52 (0, 1)	-2626<br>(1617)
Cohort received veterans' preference benefits (0, 1)	-21613<br>(1497)
Cohort identifies as male (0, 1) *x Cohort identifies as White* (0, 1)	861<br>(2117)
Cohort identifies as male (0, 1) *x Cohort born from 1950-52* (0, 1)	1043<br>(2286)
Cohort identifies as White (0, 1) *x Cohort born from 1950-52* (0, 1)	3245<br>(2286)
Cohort identifies as male (0, 1) *x Cohort received veterans' preference benefits* (0, 1)	23879<br>(2117)
Cohort identifies as White (0, 1) *x Cohort received veterans' preference benefits* (0, 1)	-47459<br>(2117)
Cohort born from 1950-52 (0, 1) *x Cohort received veterans' preference benefits* (0, 1)	2440<br>(2286)
Cohort identifies as male (0, 1) *x Cohort identifies as White* (0, 1) *x Cohort born from 1950-52* (0, 1)	2318<br>(3233)
Cohort identifies as male (0, 1) *x Cohort identifies as White* (0, 1) *x Cohort received vet' pref. benefits* (0, 1)	11686<br>(2994)
Cohort identifies as male (0, 1) *x Cohort born from 1950-52* (0, 1) *x Cohort received vet' pref. benefits* (0, 1)	149<br>(3233)
Cohort identifies as White (0, 1) *x Cohort born from 1950-52* (0, 1) *x Cohort received vet' pref. benefits* (0, 1)	3142<br>(3233)
Identifies as male (0, 1) *x Identifies as White* (0, 1) *x Born '50-'52* (0, 1) *x Received vet' pref.* (0, 1)	10834<br>(4572)
R^2	0.9948
N (Unit: Birth Cohort-Gender-Race-Veterans-Preference Grouping)	56

Note. Estimates stem from the regression of number of employees in birth cohort t, Y_t, on the variables and interactions appearing in the left hand column. Data come from the population, thus standard errors serve as a metric of variability across annual birth cohorts and do not offer a measure of sampling variation. Values are rounded.

Table S5.
Proportion of veterans' preference recipients across occupational categories

	1950 Birth Cohort		1951 Birth Cohort		1952 Birth Cohort		1953 Birth Cohort		1954 Birth Cohort		1955 Birth Cohort		1956 Birth Cohort	
Occupational Category	No VP	VP	No VP	VP	No VP	VP	No VP	VP	No VP	VP	No VP	VP	No VP	VP
Administrative	0.22	0.15	0.21	0.13	0.19	0.13	0.18	0.12	0.17	0.1	0.16	0.09	0.15	0.1
Blue Collar	0.19	0.43	0.19	0.44	0.19	0.44	0.2	0.46	0.2	0.47	0.21	0.49	0.21	0.49
Clerical	0.07	0.11	0.07	0.12	0.08	0.12	0.08	0.13	0.09	0.14	0.09	0.14	0.09	0.13
Other White Collar	0.02	0.06	0.02	0.07	0.03	0.07	0.03	0.08	0.03	0.08	0.04	0.08	0.06	0.09
Professional	0.35	0.09	0.35	0.08	0.34	0.08	0.33	0.07	0.32	0.05	0.31	0.04	0.3	0.04
Technical	0.15	0.17	0.16	0.16	0.18	0.16	0.18	0.15	0.19	0.15	0.19	0.15	0.19	0.14

Note. The table presents the proportion of non-recipients of veterans' preference and recipients of veterans' preference, respectively, across occupational categories in their first year of civilian, federal employment.

Table S6.
Proportion of veterans' preference recipients across supervisory status

	1950 Birth Cohort		1951 Birth Cohort		1952 Birth Cohort		1953 Birth Cohort		1954 Birth Cohort		1955 Birth Cohort		1956 Birth Cohort	
Supervisory Status	No VP	VP	No VP	VP	No VP	VP	No VP	VP	No VP	VP	No VP	VP	No VP	VP
Supervisory	0.17	0.12	0.16	0.11	0.14	0.10	0.13	0.09	0.11	0.08	0.11	0.08	0.10	0.08
Non-supervisory	0.83	0.88	0.84	0.89	0.86	0.90	0.87	0.91	0.89	0.92	0.89	0.92	0.90	0.92

Note. The table presents the proportion of non-recipients of veterans' preference and recipients of veterans' preference, respectively, across supervisory status in their first year of civilian, federal employment.

Table S7.
Pay differences between preference recipients and non-recipients

Year of Employment	Matching Combination 1	Matching Combination 2	Matching Combination 3	Matching Combination 4
2	0.0019	0.0023	0.0013	-0.0017
3	0.0022	0.0025	0.0011	-0.004
4	-0.00003	-0.0005	-0.0013	-0.0066
5	-0.0002	-0.003	-0.0025	-0.0087
6	-0.0009	-0.0027	-0.0031	-0.0085
7	-0.0019	-0.0045	-0.0042	-0.0099
8	-0.0005	-0.0035	-0.0025	-0.0077
9	0.0005	-0.0026	-0.001	-0.0061
10	0.0001	-0.0018	-0.0008	-0.0055
Matching Variables				
First Agency	X	X		X
First Duty Station	X		X	X
First Income	X	X	X	X
First Occupation	X	X	X	
First Year	X	X	X	X

Note. The value in each cell indicates the difference in the logarithm of mean inflation-adjusted pay between veterans' preference recipients and non-recipients matched on the covariates listed in the final five rows of the table. Given that we study the population of data, these estimates are not subject to sampling variation.

Table S8.

(A) Proportion of veterans' preference recipients identifying as Black across occupational categories

	1950 Birth Cohort		1951 Birth Cohort		1952 Birth Cohort		1953 Birth Cohort		1954 Birth Cohort		1955 Birth Cohort		1956 Birth Cohort	
Occupational Category	No VP	VP	No VP	VP	No VP	VP	No VP	VP	No VP	VP	No VP	VP	No VP	VP
Administrative	0.17	0.07	0.15	0.06	0.13	0.06	0.12	0.05	0.11	0.04	0.1	0.04	0.1	0.04
Blue Collar	0.36	0.51	0.35	0.52	0.37	0.52	0.36	0.54	0.36	0.54	0.35	0.55	0.33	0.54
Clerical	0.15	0.19	0.17	0.2	0.18	0.21	0.19	0.21	0.2	0.21	0.2	0.22	0.2	0.22
Other White Collar	0.03	0.06	0.03	0.07	0.03	0.06	0.04	0.06	0.05	0.07	0.08	0.06	0.11	0.07
Professional	0.16	0.02	0.14	0.02	0.14	0.02	0.13	0.01	0.13	0.01	0.11	0.01	0.11	0.01
Technical	0.15	0.14	0.16	0.14	0.15	0.14	0.16	0.12	0.15	0.12	0.15	0.12	0.14	0.12

Note. The table presents the proportion of non-recipients of veterans' preference and recipients of veterans' preference, respectively, who identify as Black, across occupational categories in their first year of civilian, federal employment.

(B) Proportion of veterans' preference recipients identifying as White across occupational categories

	1950 Birth Cohort		1951 Birth Cohort		1952 Birth Cohort		1953 Birth Cohort		1954 Birth Cohort		1955 Birth Cohort		1956 Birth Cohort	
Occupational Category	No VP	VP	No VP	VP	No VP	VP	No VP	VP	No VP	VP	No VP	VP	No VP	VP
Administrative	0.23	0.16	0.21	0.15	0.2	0.14	0.18	0.13	0.17	0.13	0.16	0.12	0.16	0.13
Blue Collar	0.16	0.41	0.16	0.42	0.17	0.42	0.18	0.43	0.19	0.44	0.19	0.46	0.2	0.47
Clerical	0.06	0.09	0.06	0.1	0.07	0.11	0.07	0.11	0.08	0.11	0.08	0.11	0.08	0.1
Other White Collar	0.02	0.06	0.02	0.07	0.02	0.07	0.03	0.08	0.03	0.08	0.04	0.09	0.05	0.09
Professional	0.37	0.1	0.36	0.09	0.35	0.09	0.34	0.09	0.33	0.07	0.32	0.05	0.31	0.06
Technical	0.17	0.18	0.18	0.18	0.2	0.17	0.2	0.16	0.2	0.17	0.2	0.17	0.2	0.16

Note. The table presents the proportion of non-recipients of veterans' preference and recipients of veterans' preference, respectively, who identify as White, across occupational categories in their first year of civilian, federal employment.

Table S9.

(A) Proportion of veterans' preference recipients identifying as Black across supervisory status

	1950 Birth Cohort		1951 Birth Cohort		1952 Birth Cohort		1953 Birth Cohort		1954 Birth Cohort		1955 Birth Cohort		1956 Birth Cohort	
Supervisory Status	No VP	VP	No VP	VP	No VP	VP	No VP	VP	No VP	VP	No VP	VP	No VP	VP
Supervisory	0.12	0.09	0.11	0.08	0.1	0.07	0.1	0.06	0.08	0.06	0.08	0.05	0.07	0.06
Non-supervisory	0.88	0.91	0.89	0.92	0.9	0.93	0.9	0.94	0.92	0.94	0.92	0.95	0.93	0.94

Note. The table presents the proportion of non-recipients of veterans' preference and recipients of veterans' preference, respectively, who identify as Black, across supervisory status in their first year of civilian, federal employment.

(B) Proportion of veterans' preference recipients identifying as White across supervisory status

	1950 Birth Cohort		1951 Birth Cohort		1952 Birth Cohort		1953 Birth Cohort		1954 Birth Cohort		1955 Birth Cohort		1956 Birth Cohort	
Supervisory Status	No VP	VP	No VP	VP	No VP	VP	No VP	VP	No VP	VP	No VP	VP	No VP	VP
Supervisory	0.09	0.14	0.18	0.12	0.16	0.11	0.14	0.11	0.13	0.09	0.12	0.09	0.11	0.09
Non-supervisory	0.81	0.86	0.82	0.88	0.84	0.89	0.86	0.89	0.87	0.91	0.88	0.91	0.89	0.91

Note. The table presents the proportion of non-recipients of veterans' preference and recipients of veterans' preference, respectively, who identify as White, across supervisory status in their first year of civilian, federal employment.

Table S10.

(A)
Pay differences between preference recipients and non-recipients identifying as Black

Year of Employment	Matching Combination 1	Matching Combination 2	Matching Combination 3	Matching Combination 4
2	0.004583	0.001658	0.003435	-0.0029
3	0.000797	-0.00209	0.000273	-0.00588
4	0.001306	-0.00029	0.001171	-0.00617
5	0.003531	0.003779	0.001544	-0.00694
6	-0.00321	-0.00223	-0.00261	-0.01016
7	-0.00322	-0.00028	-0.00253	-0.00894
8	-2.30×10^{-5}	4.40×10^{-5}	0.001376	-0.00581
9	-0.00274	-0.002	-0.00182	-0.00689
10	0.001849	0.001182	0.003247	-0.00706
Matching Variables				
First Agency	X	X		X
First Duty Station	X		X	X
First Income	X	X	X	X
First Occupation	X	X	X	
First Year	X	X	X	X

(B)
Pay differences between preference recipients and non-recipients identifying as Black

Year of Employment	Matching Combination 1	Matching Combination 2	Matching Combination 3	Matching Combination 4
2	0.001493	0.001151	0.001042	-0.00168
3	0.002537	0.002285	0.001757	-0.00282
4	0.001187	0.000612	2.00×10^{-6}	-0.00646
5	-0.0011	-0.00249	-0.00205	-0.00902
6	0.000423	-0.00135	-0.00092	-0.00666
7	-0.00279	-0.0038	-0.00405	-0.00957
8	-0.00104	-0.00253	-0.00232	-0.00762
9	-0.00043	-0.00117	-0.00173	-0.00728
10	-0.00219	-0.00116	-0.003	-0.00617
Matching Variables				
First Agency	X	X		X
First Duty Station	X		X	X
First Income	X	X	X	X
First Occupation	X	X	X	
First Year	X	X	X	X

Note. The value in each cell indicates the difference in the logarithm of mean inflation-adjusted pay between veterans' preference recipients and non-recipients matched on the variables listed in the final five rows of the table. Given that we study the population of data, these estimates are not subject to sampling variation.

Table S11.
Proportion of non-Recipients and recipients of veterans' preference by identity group (no DoD)

	1950 Birth Cohort		1951 Birth Cohort		1952 Birth Cohort		1953 Birth Cohort		1954 Birth Cohort		1955 Birth Cohort		1956 Birth Cohort	
Racial or National Identity Group	No VP	VP	No VP	VP	No VP	VP	No VP	VP	No VP	VP	No VP	VP	No VP	VP
American Indian or Alaskan Native	0.02	0.02	0.02	0.01	0.02	0.02	0.02	0.01	0.02	0.01	0.02	0.01	0.02	0.01
Asian or Pacific Islander	0.04	0.03	0.04	0.03	0.04	0.03	0.04	0.03	0.04	0.03	0.04	0.02	0.04	0.02
Black, not of Hispanic Origin	0.09	0.18	0.09	0.19	0.09	0.21	0.09	0.23	0.1	0.25	0.11	0.26	0.11	0.24
Hispanic	0.04	0.07	0.04	0.08	0.04	0.07	0.05	0.08	0.05	0.08	0.05	0.09	0.05	0.08
Not Hispanic in Puerto Rico	≈0	≈0	≈0	≈0	≈0	≈0	≈0	≈0	≈0	≈0	≈0	≈0	≈0	≈0
White, not of Hispanic Origin	0.8	0.71	0.8	0.68	0.81	0.67	0.8	0.65	0.79	0.63	0.78	0.62	0.78	0.64

Note. The table displays, for each birth cohort in the VSSL, the proportion of veterans' preference non-recipients and recipients, respectively, identifying with each of the racial or national identity groups listed in the left hand margin of the table (excluding employees in the Department of Defense [DoD]). The proportions of non-recipients across identity groups, in each birth cohort, appears under the column heading "No VP." The proportions of recipients across identity groups, in each birth cohort, appears under the column heading "VP." Thus, the denominator for each of the decimal values in, say, the column labeled "No VP," under the heading "1950 Birth Cohort," is the total number of employees in the 1950 birth cohort who did not receive veterans' preference benefits and the numerator is the number of said individuals identifying with the racial or national identity group in the corresponding left-hand margin.

Table S12.
Proportion of identity group by receipt of veterans' preference (no DoD)

	1950 Birth Cohort		1951 Birth Cohort		1952 Birth Cohort		1953 Birth Cohort		1954 Birth Cohort		1955 Birth Cohort		1956 Birth Cohort	
Racial or National Identity Group	No VP	VP	No VP	VP	No VP	VP	No VP	VP	No VP	VP	No VP	VP	No VP	VP
American Indian or Alaskan Native	0.55	0.45	0.65	0.35	0.63	0.37	0.69	0.31	0.72	0.28	0.75	0.25	0.8	0.2
Asian or Pacific Islander	0.59	0.41	0.62	0.38	0.61	0.39	0.65	0.35	0.69	0.31	0.74	0.26	0.79	0.21
Black, not of Hispanic Origin	0.34	0.66	0.38	0.62	0.36	0.64	0.37	0.63	0.39	0.61	0.42	0.58	0.52	0.48
Hispanic, not identifying as White	0.38	0.62	0.41	0.59	0.43	0.57	0.47	0.53	0.49	0.51	0.52	0.48	0.61	0.39
Not Hispanic in Puerto Rico	0.59	0.41	0.67	0.33	0.71	0.29	0.77	0.23	0.74	0.26	0.71	0.29	0.7	0.3
White, not of Hispanic Origin	0.53	0.47	0.6	0.4	0.6	0.4	0.64	0.36	0.67	0.33	0.7	0.3	0.74	0.26

Note. The table displays, for each birth cohort in the VSSL, the proportion of each racial or national identity group that did not receive and did receive veterans' preference benefits (excluding employees in the Department of Defense [DoD]). The proportions of non-recipients across identity groups, in each birth cohort, appears under the column heading "No VP." The proportions of recipients across identity groups, in each birth cohort, appears under the column heading "VP." Thus, the denominator for each of the decimal values in, say, the column labeled "No VP," under the heading "1950 Birth Cohort," in the row labeled "White, Not of Hispanic Origin" is the total number of employees in the 1950 birth cohort who identified as White, Not Hispanic, and the numerator is the number of said individuals who did not receive veterans' preference. Values are rounded.

Table S13.
Estimates from difference-in-difference-in-difference model (no DoD)

Parameter	Estimate
Intercept	24296<br>(1143)
Cohort identifies as male (0, 1)	-5936<br>(1616)
Cohort identifies as White (0, 1)	52056<br>(1616)
Cohort born from 1950-52 (0, 1)	-2674<br>(1745)
Cohort identifies as male (0, 1) *x Cohort identifies as White* (0, 1)	15289<br>(2285)
Cohort identifies as male (0, 1) *x Cohort born from 1950-52* (0, 1)	2107<br>(2468)
Cohort identifies as White (0, 1) *x Cohort born from 1950-52* (0, 1)	-3571<br>(2468)
Cohort identifies as male (0, 1) *x Cohort identifies as White* (0, 1) *x Cohort born from 1950-52* (0, 1)	16399<br>(3491)
R^2	0.99
N (Unit: Birth Cohort-Gender-Race Grouping)	28

Note. Estimates stem from the regression of number of employees in birth cohort t, Y_t, on the variables and interactions appearing in the left hand column. Data exclude employees from the Department of Defense [DoD] and come from the population, thus standard errors serve as a metric of variability across annual birth cohorts and do not offer a measure of sampling variation. Values are rounded.

Table S14.
Estimates from diff.-in-diff.-in-diff.-in-diff. model (no DoD)

Parameter	Estimate
Intercept	22496<br>(1030)
Cohort identifies as male (0, 1)	-14425<br>(1457)
Cohort identifies as White (0, 1)	48208<br>(1457)
Cohort born from 1950-52 (0, 1)	-2496<br>(1573)
Cohort received veterans' preference benefits (0, 1)	-20747<br>(1457)
Cohort identifies as male (0, 1) *x Cohort identifies as White* (0, 1)	2573<br>(2060)
Cohort identifies as male (0, 1) *x Cohort born from 1950-52* (0, 1)	989<br>(2225)
Cohort identifies as White (0, 1) *x Cohort born from 1950-52* (0, 1)	-3390<br>(2225)
Cohort identifies as male (0, 1) *x Cohort received veterans' preference benefits* (0, 1)	22895<br>(2060)
Cohort identifies as White (0, 1) *x Cohort received veterans' preference benefits* (0, 1)	-44683<br>(2060)
Cohort born from 1950-52 (0, 1) *x Cohort received veterans' preference benefits* (0, 1)	2327<br>(2225)
Cohort identifies as male (0, 1) *x Cohort identifies as White* (0, 1) *x Cohort born from 1950-52* (0, 1)	2508<br>(3147)
Cohort identifies as male (0, 1) *x Cohort identifies as White* (0, 1) *x Cohort received vets' pref. benefits* (0, 1)	9713<br>(2913)
Cohort identifies as male (0, 1) *x Cohort born from 1950-52* (0, 1) *x Cohort received vets' pref. benefits* (0, 1)	97<br>(3147)
Cohort identifies as White (0, 1) *x Cohort born from 1950-52* (0, 1) *x Cohort received vets' pref. benefits* (0, 1)	3269<br>(3147)
Identifies as male (0, 1) *x Identifies as White* (0, 1) *x Born '50-'52* (0, 1) *x Received vets' pref.* (0, 1)	10244<br>(4450)
R^2	0.99
N (Unit: Birth Cohort-Gender-Race-Veterans-Preference Grouping)	56

Note. Estimates stem from the regression of number of employees in birth cohort t, Y_t, on the variables and interactions appearing in the left hand column. Data exclude employees in the Department of Defense [DoD] and come from the population, thus standard errors serve as a metric of variability across annual birth cohorts and do not offer a measure of sampling variation. Values are rounded.

Table S15.
Proportion of veterans' preference recipients across occup. categories (no DoD)

	1950 Birth Cohort		1951 Birth Cohort		1952 Birth Cohort		1953 Birth Cohort		1954 Birth Cohort		1955 Birth Cohort		1956 Birth Cohort	
Occupational Category	No VP	VP	No VP	VP	No VP	VP	No VP	VP	No VP	VP	No VP	VP	No VP	VP
Administrative	0.21	0.13	0.19	0.12	0.18	0.11	0.16	0.1	0.15	0.09	0.14	0.09	0.13	0.1
Blue Collar	0.19	0.44	0.19	0.45	0.2	0.45	0.21	0.46	0.22	0.48	0.22	0.49	0.22	0.49
Clerical	0.07	0.11	0.08	0.13	0.08	0.14	0.09	0.15	0.09	0.15	0.09	0.15	0.1	0.14
Other White Collar	0.02	0.06	0.02	0.07	0.03	0.07	0.04	0.08	0.04	0.08	0.06	0.08	0.08	0.09
Professional	0.34	0.08	0.33	0.07	0.32	0.07	0.3	0.06	0.29	0.05	0.28	0.04	0.27	0.04
Technical	0.17	0.17	0.19	0.16	0.2	0.16	0.2	0.15	0.2	0.16	0.2	0.15	0.19	0.15

Note. The table presents the proportion of non-recipients of veterans' preference and recipients of veterans' preference, respectively, across occupational categories in their first year of civilian, federal employment. The data exclude employees working in the Department of Defense [DoD]. Values are rounded.

Table S16.
Proportion of veterans' preference recipients across supervisory status (no DoD)

	1950 Birth Cohort		1951 Birth Cohort		1952 Birth Cohort		1953 Birth Cohort		1954 Birth Cohort		1955 Birth Cohort		1956 Birth Cohort	
Supervisory Status	No VP	VP	No VP	VP	No VP	VP	No VP	VP	No VP	VP	No VP	VP	No VP	VP
Supervisory	0.17	0.12	0.16	0.1	0.14	0.1	0.13	0.09	0.11	0.08	0.1	0.07	0.10	0.08
Non-supervisory	0.83	0.88	0.84	0.9	0.86	0.9	0.87	0.91	0.89	0.92	0.9	0.93	0.9	0.92

Note. The table presents the proportion of non-recipients of veterans' preference and recipients of veterans' preference, respectively, across supervisory status in their first year of civilian, federal employment. The data exclude employees working in the Department of Defense [DoD]. Values are rounded.

Table S17.
Pay differences between preference recipients and non-recipients (no DoD)

Year of Employment	Matching Combination 1	Matching Combination 2	Matching Combination 3	Matching Combination 4
2	0.0022	0.002197	0.0015	-0.0015
3	0.0026	0.002954	0.0015	-0.0039
4	0.001	0.000522	-5.00×10^{-4}	-0.0065
5	2.00×10^{-4}	-0.00207	-0.0021	-0.0094
6	-0.0015	-0.00283	-0.0035	-0.0094
7	-0.002	-0.00474	-0.0041	-0.0099
8	-0.0016	-0.00433	-0.0033	-0.0083
9	-0.0011	-0.00276	-0.0029	-0.0073
10	-4.00×10^{-4}	-0.0017	-0.001	-0.0055
Matching Variables				
First Agency	X	X		X
First Duty Station	X		X	X
First Income	X	X	X	X
First Occupation	X	X	X	
First Year	X	X	X	X

Note. The value in each cell indicates the difference in the logarithm of mean inflation-adjusted pay between veterans' preference recipients and non-recipients matched on the variables listed in the final five rows of the table. Given that we study the population of data, these estimates are not subject to sampling variation. The data exclude employees working in the Department of Defense [DoD]. Values are rounded.

Table S18.

(A) Proportion of veterans' preference recipients identifying as Black across occupational categories (no DoD)

	1950 Birth Cohort		1951 Birth Cohort		1952 Birth Cohort		1953 Birth Cohort		1954 Birth Cohort		1955 Birth Cohort		1956 Birth Cohort	
Occupational Category	No VP	VP	No VP	VP	No VP	VP	No VP	VP	No VP	VP	No VP	VP	No VP	VP
Administrative	0.17	0.07	0.15	0.06	0.13	0.06	0.12	0.05	0.11	0.04	0.1	0.04	0.1	0.04
Blue Collar	0.36	0.51	0.35	0.52	0.37	0.52	0.36	0.54	0.36	0.54	0.35	0.55	0.33	0.54
Clerical	0.15	0.19	0.17	0.2	0.18	0.21	0.19	0.21	0.2	0.21	0.2	0.22	0.2	0.22
Other White Collar	0.03	0.06	0.03	0.07	0.03	0.06	0.04	0.06	0.05	0.07	0.08	0.06	0.11	0.07
Professional	0.16	0.02	0.14	0.02	0.14	0.02	0.13	0.01	0.13	0.01	0.11	0.01	0.11	0.01
Technical	0.15	0.14	0.16	0.14	0.15	0.14	0.16	0.12	0.15	0.12	0.15	0.12	0.14	0.12

Note. The table presents the proportion of non-recipients of veterans' preference and recipients of veterans' preference, respectively, across occupational categories in their first year of civilian, federal employment. The data exclude employees working in the Department of Defense [DoD]. Values are rounded.

(B) Proportion of veterans' preference recipients identifying as White across occupational categories (no DoD)

	1950 Birth Cohort		1951 Birth Cohort		1952 Birth Cohort		1953 Birth Cohort		1954 Birth Cohort		1955 Birth Cohort		1956 Birth Cohort	
Occupational Category	No VP	VP	No VP	VP	No VP	VP	No VP	VP	No VP	VP	No VP	VP	No VP	VP
Administrative	0.23	0.16	0.21	0.15	0.2	0.14	0.18	0.13	0.17	0.13	0.16	0.12	0.16	0.13
Blue Collar	0.16	0.41	0.16	0.42	0.17	0.42	0.18	0.43	0.19	0.44	0.19	0.46	0.2	0.47
Clerical	0.06	0.09	0.06	0.1	0.07	0.11	0.07	0.11	0.08	0.11	0.08	0.11	0.08	0.1
Other White Collar	0.02	0.06	0.02	0.07	0.02	0.07	0.03	0.08	0.03	0.08	0.04	0.09	0.05	0.09
Professional	0.37	0.1	0.36	0.09	0.35	0.09	0.34	0.09	0.33	0.07	0.32	0.05	0.31	0.06
Technical	0.17	0.18	0.18	0.18	0.2	0.17	0.2	0.16	0.2	0.17	0.2	0.17	0.2	0.16

Note. The table presents the proportion of non-recipients of veterans' preference and recipients of veterans' preference, respectively, across occupational categories in their first year of civilian, federal employment. The data exclude employees working in the Department of Defense [DoD]. Values are rounded.

Table S19.

(A) Proportion of veterans' preference recipients identifying as Black across supervisory status (no DoD)

	1950 Birth Cohort		1951 Birth Cohort		1952 Birth Cohort		1953 Birth Cohort		1954 Birth Cohort		1955 Birth Cohort		1956 Birth Cohort	
Supervisory Status	No VP	VP	No VP	VP	No VP	VP	No VP	VP	No VP	VP	No VP	VP	No VP	VP
Supervisory	0.12	0.09	0.11	0.08	0.1	0.07	0.1	0.06	0.08	0.06	0.08	0.05	0.07	0.06
Non-supervisory	0.88	0.91	0.89	0.92	0.9	0.93	0.9	0.94	0.92	0.94	0.92	0.95	0.93	0.94

Note. The table presents the proportion of non-recipients of veterans' preference and recipients of veterans' preference, respectively, across supervisory status in their first year of civilian, federal employment. The data exclude employees working in the Department of Defense [DoD]. Values are rounded.

(B) Proportion of veterans' preference recipients identifying as White across supervisory status (no DoD)

	1950 Birth Cohort		1951 Birth Cohort		1952 Birth Cohort		1953 Birth Cohort		1954 Birth Cohort		1955 Birth Cohort		1956 Birth Cohort	
Supervisory Status	No VP	VP	No VP	VP	No VP	VP	No VP	VP	No VP	VP	No VP	VP	No VP	VP
Supervisory	0.09	0.14	0.18	0.12	0.16	0.11	0.14	0.11	0.13	0.09	0.12	0.09	0.11	0.09
Non-supervisory	0.81	0.86	0.82	0.88	0.84	0.89	0.86	0.89	0.87	0.91	0.88	0.91	0.89	0.91

Note. The table presents the proportion of non-recipients of veterans' preference and recipients of veterans' preference, respectively, across supervisory status in their first year of civilian, federal employment. The data exclude employees working in the Department of Defense [DoD]. Values are rounded.

Table S20.

(A)
Pay differences between preference recipients and non-recipients identifying as Black (no DoD)

Year of Employment	Matching Combination 1	Matching Combination 2	Matching Combination 3	Matching Combination 4
2	0.004583	0.001658	0.003435	-0.0029
3	0.000797	-0.00209	0.000273	-0.00588
4	0.001306	-0.00029	0.001171	-0.00617
5	0.003531	0.003779	0.001544	-0.00694
6	-0.00321	-0.00223	-0.00261	-0.01016
7	-0.00322	-0.00028	-0.00253	-0.00894
8	-2.30×10^{-5}	4.40×10^{-5}	0.001376	-0.00581
9	-0.00274	-0.002	-0.00182	-0.00689
10	0.001849	0.001182	0.003247	-0.00706
Matching Variables				
First Agency	X	X		X
First Duty Station	X		X	X
First Income	X	X	X	X
First Occupation	X	X	X	
First Year	X	X	X	X

(B)
Pay differences between preference recipients and non-recipients identifying as White (no DoD)

Year of Employment	Matching Combination 1	Matching Combination 2	Matching Combination 3	Matching Combination 4
2	0.001493	0.001151	0.001042	-0.00168
3	0.002537	0.002285	0.001757	-0.00282
4	0.001187	0.000612	2.00×10^{-6}	-0.00646
5	-0.0011	-0.00249	-0.00205	-0.00902
6	0.000423	-0.00135	-0.00092	-0.00666
7	-0.00279	-0.0038	-0.00405	-0.00957
8	-0.00104	-0.00253	-0.00232	-0.00762
9	-0.00043	-0.00117	-0.00173	-0.00728
10	-0.00219	-0.00116	-0.003	-0.00617
Matching Variables				
First Agency	X	X		X
First Duty Station	X		X	X
First Income	X	X	X	X
First Occupation	X	X	X	
First Year	X	X	X	X

Note. The value in each cell indicates the difference in the logarithm of mean inflation-adjusted pay between veterans' preference recipients and non-recipients matched on the listed covariates. Given that we study the population, these estimates are not subject to sampling variation. Data exclude the Department of Defense [DoD]. Values are rounded.

37. References cited in the supplementary materials

1. Lewis GB (2013) The Impact of Veterans' Preference on the Composition and Quality of the Federal Service. Journal of Public Administration Research and Theory 23: 247-265.

2. Johnson T, Walker RW (2018) The Career Advancement of Military Veterans in Recent Cohorts of the U.S. Executive Branch. Public Personnel Management 47: 382–397.

3. Campbell A (1977). Statement at Veterans Preference Oversight Hearings. U.S. House of Representatives, Subcommittee on Civil Service of the Committee on Post Office and Civil Service. Serial No. 95-43 (Washington, D.C., U.S. Government Printing Office).

4. Lewis GB, Emmert MA. (1984) Who pays for veterans' preference? Administration & Society 16: 328-345.

5. Angrist JD (1990) Lifetime Earnings and the Vietnam Era Draft Lottery: Evidence from Social Security Administrative Records. American Economic Review 80: 313-36.

6. Wilkins VM, Keiser LR (2006) Linking Passive and Active Representation by Gender: The Case of Child Support Agencies. Journal of Public Administration Research and Theory 16: 87-102.

7. Johnson T (2015) Service after Serving: Does Veterans' Preference Diminish the Quality of the US Federal Service? Journal of Public Administration Research and Theory 25:669-696.

8. Lipsky M (1980) Street-level Bureaucracy: Dilemmas of the Individual in Public Services (New York, Russell Sage Foundation).

9. Office of Personnel Management (2007) Operating Manual: Guide to Personnel Data Standards (August 10). (Washington, D.C., Office of Personnel Management).

10. King G. 2012. An explanation for CEM weights. Available online: https://docs.google.com/document/d/1xQwyLt_6EXdNpA685LjmhjO20y5pZDZYwe2qeNoI5dE/edit
